# Supplementary material for: Synthesis of Oleanolic Acid-Dithiocarbamate Conjugates and Evaluation of Their Broad-Spectrum Antitumor Activities
Source: Molecules. 2023 Feb 2;28(3):1414. doi: 10.3390/molecules28031414 (PMC9920998; doi:10.3390/molecules28031414)
Supplement: Supplementary file 1 [file molecules-28-01414-s001.zip › molecules-2173404-supplementary.pdf]

Supplementary Information

Synthesis of oleanolic acid-dithiocarbamate conjugates and evaluation of their broad-spectrum antitumor activities

Liyao Tang,[†,§] Yan Zhang,[†,§] Jinrun Xu,[†,§] Qingfan Yang,[‡] Fukuan Du,[†,⊥] Xu Wu,[†,⊥] Mingxing Li,[†,⊥] Jing Shen,[†,⊥] Shuai Deng,[†,⊥] Yueshui Zhao,[*,†,⊥] Zhangang Xiao,[*,‡,⊥] and Yu Chen[*,†,⊥]

[†]*Laboratory of Molecular Pharmacology, Department of Pharmacology, School of Pharmacy, Southwest Medical University, Luzhou 646000, China*

[‡]*Department of Oncology, The Affiliated Hospital of Southwest Medical University, Southwest Medical University, Luzhou 646000, China*

[⊥]*Cell Therapy & Cell Drugs of Luzhou Key Laboratory, Southwest Medical University, Luzhou 646000, China*

[§]*These authors contributed equally to this work*

E-mail: * yueshuizhao@swmu.edu.cn; zhangangxiao@swmu.edu.cn; chenyu2021@swmu.edu.cn.

Table of Contents

Figure S1. ^{1}H NMR spectrum of 2……………………………………………………S6

Figure S2.^{13}C NMR spectrum of 2……………………………………………………S6

Figure S3. ^{1}H NMR spectrum of 3a…………………………………………………..S7

Figure S4.^{13}C NMR spectrum of 3a…………………………………………………..S7

Figure S5. ^{1}H NMR spectrum of 3b…………………………………………………..S8

Figure S6. ^{13}C NMR spectrum of 3b…………………………………………………S8

Figure S7. ^{1}H NMR spectrum of 3c…………………………………………………...S9

Figure S8. ^{13}C NMR spectrum of 3c…………………………………………………..S9

Figure S9. ^{1}H NMR spectrum of 3d…………………………………………………S10

Figure S10. ^{13}C NMR spectrum of 3d………………………………………………..S10

Figure S11. ^{1}H NMR spectrum of 3e…………………………………………………S11

Figure S12. ^{13}C NMR spectrum of 3e………………………………………………...S11

Figure S13. ^{1}H NMR spectrum of 3f………………………………………………....S12

Figure S14. ^{13}C NMR spectrum of 3f………………………………………………...S12

Figure S15. ^{1}H NMR spectrum of 3g………………………………………………...S13

Figure S16. ^{13}C NMR spectrum of 3g……………………………………….…………S13

Figure S17. ^{1}H NMR spectrum of 3h………………………………………………...S14

Figure S18. ^{13}C NMR spectrum of 3h……………………………………………..S14

Figure S19. ^{1}H NMR spectrum of 3i………………………………………………S15

Figure S20. ^{13}C NMR spectrum of 3i……………………………………………...S15

Figure S21. ^{1}H NMR spectrum of 3j………………………………………………S16

Figure S22. ^{13}C NMR spectrum of 3j……………………………………………...S16

Figure S23. ^{1}H NMR spectrum of 3k……………………………………………...S17

Figure S24. ^{13}C NMR spectrum of 3k……………………………………………..S17

Figure S25. ^{1}H NMR spectrum of 3l…………………………………...…………..S18

Figure S26. ^{13}C NMR spectrum of 3l…………………………………..………..S18

Figure S27. ^{1}H NMR spectrum of 3m…………………………..…………………S19

Figure S28. ^{13}C NMR spectrum of 3m……………………………………………….S19

Figure S29. ^{1}H NMR spectrum of 3n……………………………………………...S20

Figure S30. ^{13}C NMR spectrum of 3n………………………………….……………S20

Figure S31. ^{1}H NMR spectrum of 3o…………………………...………………S21

Figure S32. ^{13}C NMR spectrum of 3o……………………………………………..S21

Figure S33. ^{1}H NMR spectrum of 3p……………………………………………...S22

Figure S34. ^{13}C NMR spectrum of 3p…………………………..………………S22

Figure S35. ^{1}H NMR spectrum of 3q…………………………..…………………S23

Figure S36. ^{13}C NMR spectrum of 3q……………………………………..…………S23

Figure S37. ^{1}H NMR spectrum of 3r……………………………………..…….…….S24

Figure S38. ^{13}C NMR spectrum of 3r…………………………………………………...S24

Figure S39. ^{1}H NMR spectrum of 3s………………………………………………….S25

Figure S40. ^{13}C NMR spectrum of 3s…….……………………………………………..S25

Figure S41. ^{1}H NMR spectrum of 3t……………………………………………………S26

Figure S42. ^{13}C NMR spectrum of 3t……………………………..……………….S26

Figure S43. HRMS spectrum of 2……………………………..………………… S27

Figure S44. HRMS spectrum of 3a……………………………..…………………S27

Figure S45. HRMS spectrum of 3b……………………………..…………………S28

Figure S46. HRMS spectrum of 3c……………………………..…………………S28

Figure S47. HRMS spectrum of 3d……………………………..…………………S29

Figure S48. HRMS spectrum of 3e……………………………..…………………S29

Figure S49. HRMS spectrum of 3f……………………………..…………………S30

Figure S50. HRMS spectrum of 3g……………………………..…………………S30

Figure S51. HRMS spectrum of 3h……………………………..…………………S31

Figure S52. HRMS spectrum of 3i……………………………..…………………S31

Figure S53. HRMS spectrum of 3j……………………………..…………………S32

Figure S54. HRMS spectrum of 3k……………………………..…………………S32

Figure S55. HRMS spectrum of 3l……………………………..…………………S33

Figure S56. HRMS spectrum of 3m……………………………..……………….S33

Figure S57. HRMS spectrum of 3n……………………………..…………………S34

Figure S58. HRMS spectrum of 3o……………………………..…………………S34

Figure S59. HRMS spectrum of 3p……………………………..…………………S35

Figure S60. HRMS spectrum of 3q……………………………..…………………S35

Figure S61. HRMS spectrum of 3r……………………………..…………………S36

Figure S62. HRMS spectrum of 3s……………………………..…………………S36

Figure S63. HRMS spectrum of 3t……………………………..…………………S37

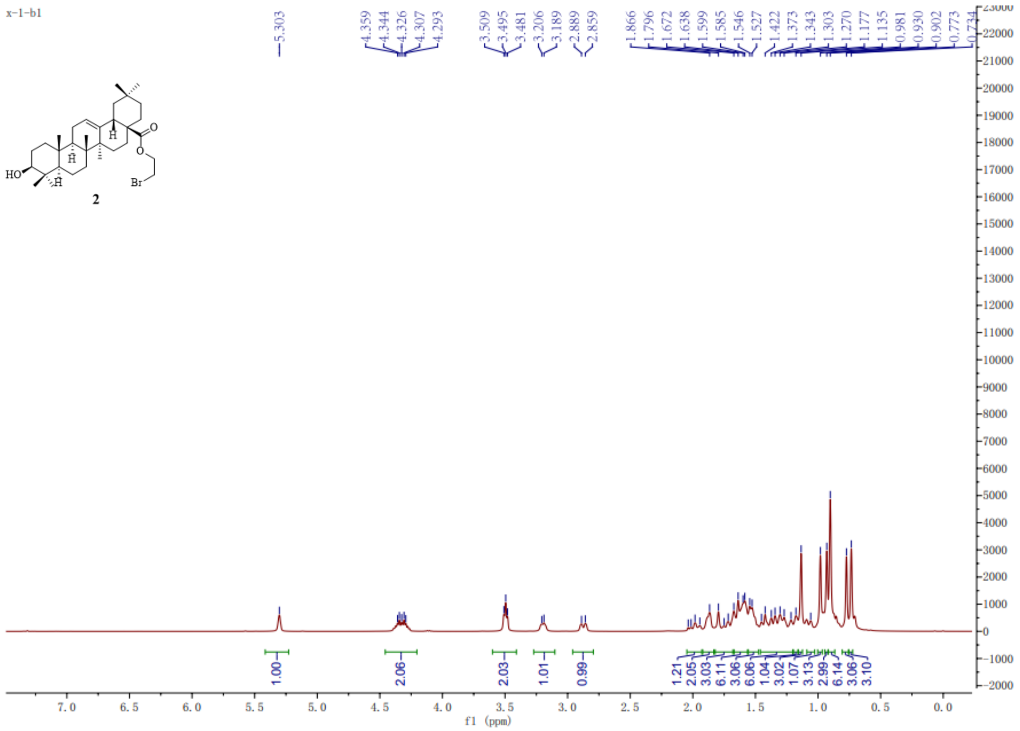

Figure S1. **^{1}H NMR spectrum of 2**.

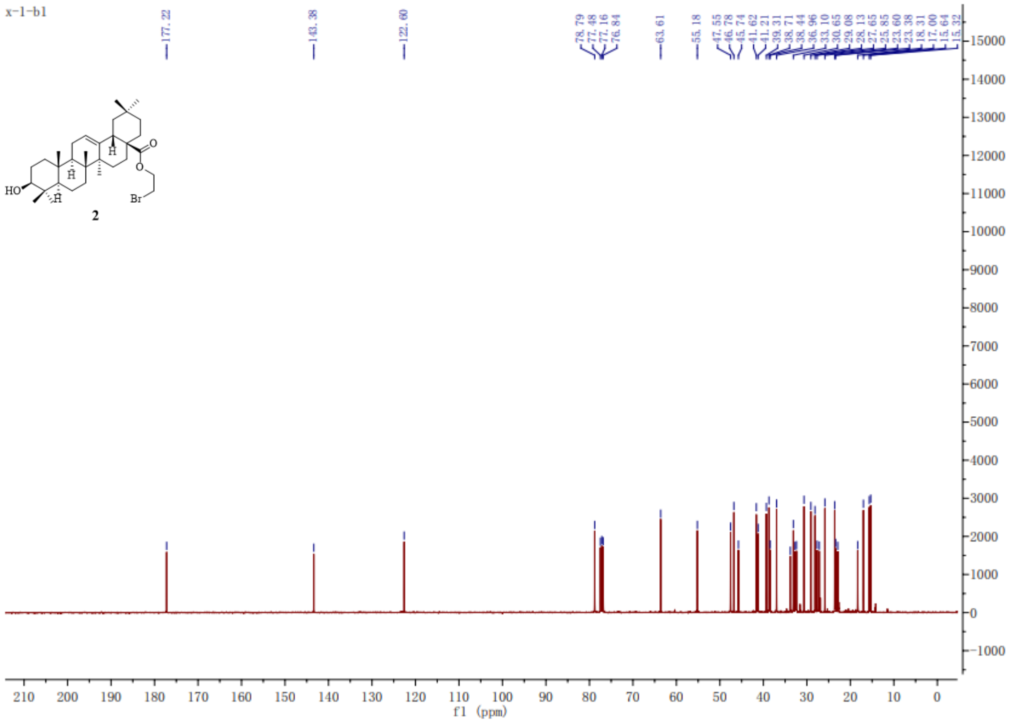

Figure S2. **^{13}C NMR spectrum of 2**.

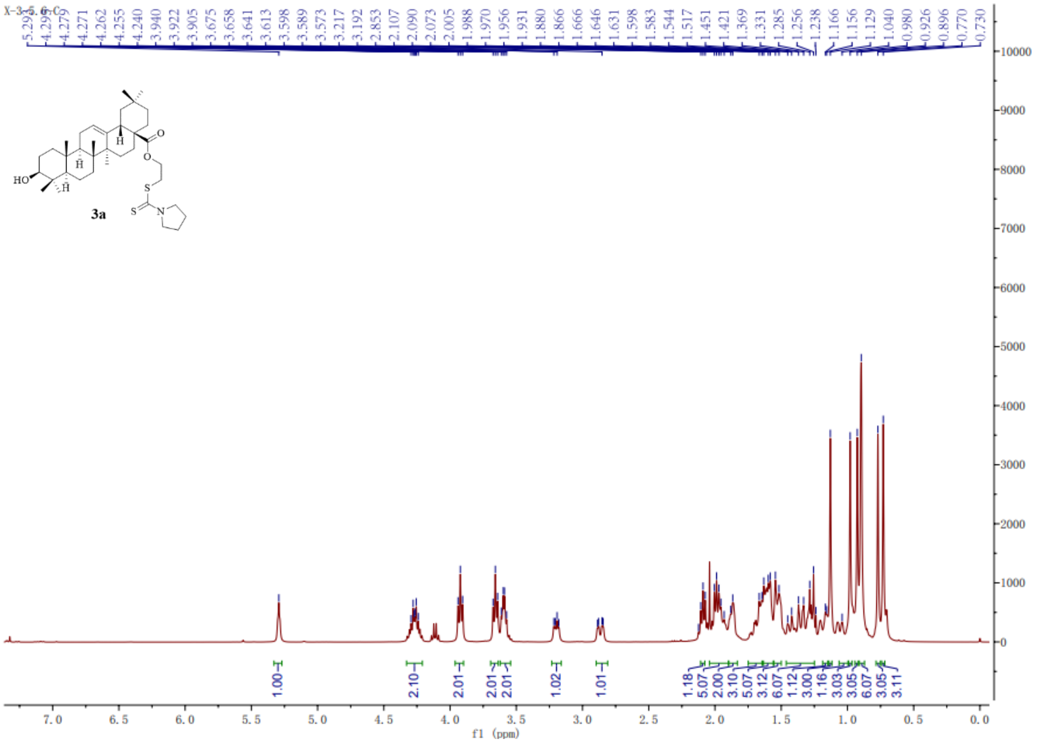

Figure S3. ^{1}H NMR spectrum of 3a.

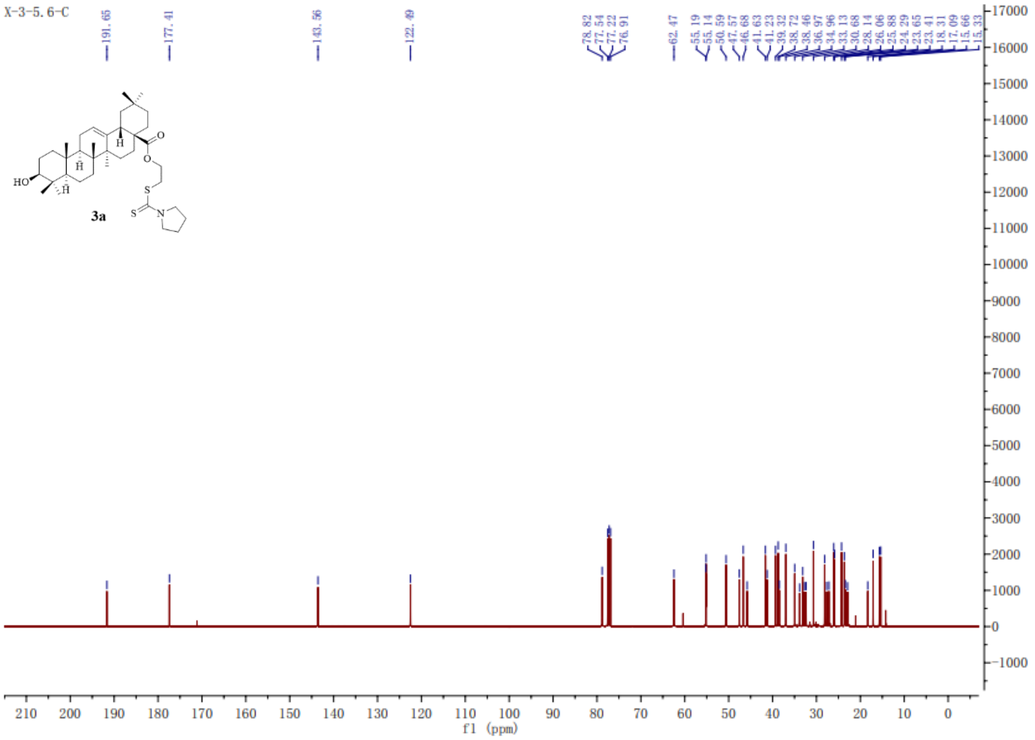

Figure S4. ^{13}C NMR spectrum of 3a.

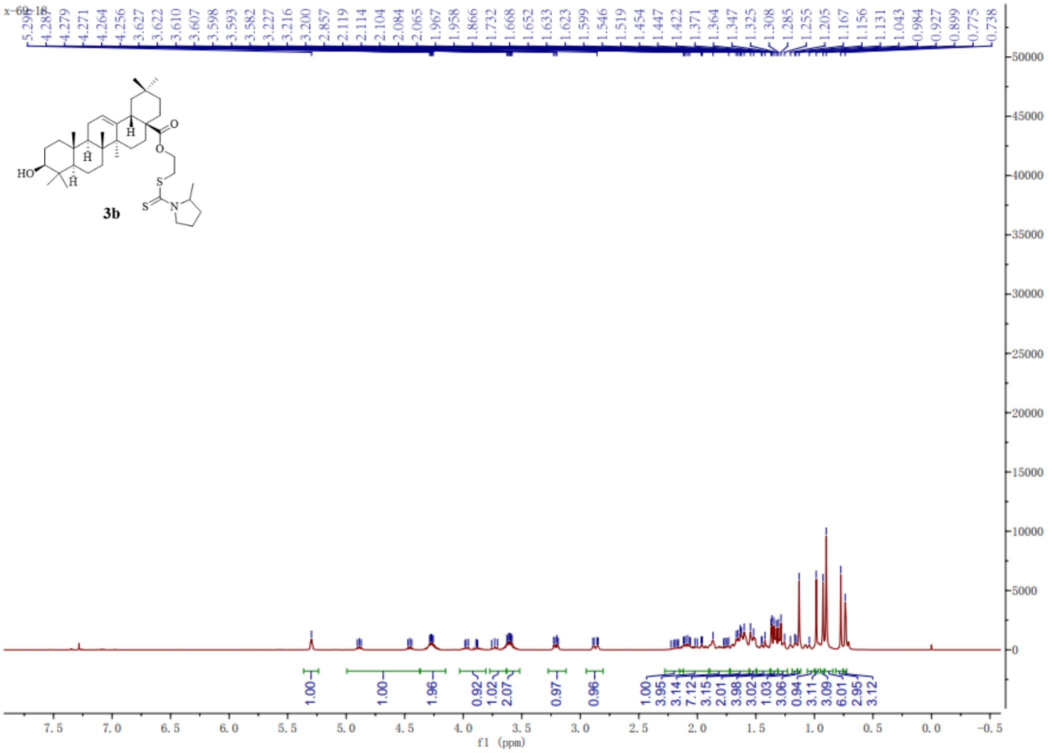

Figure S5. ^{1}H NMR spectrum of 3b.

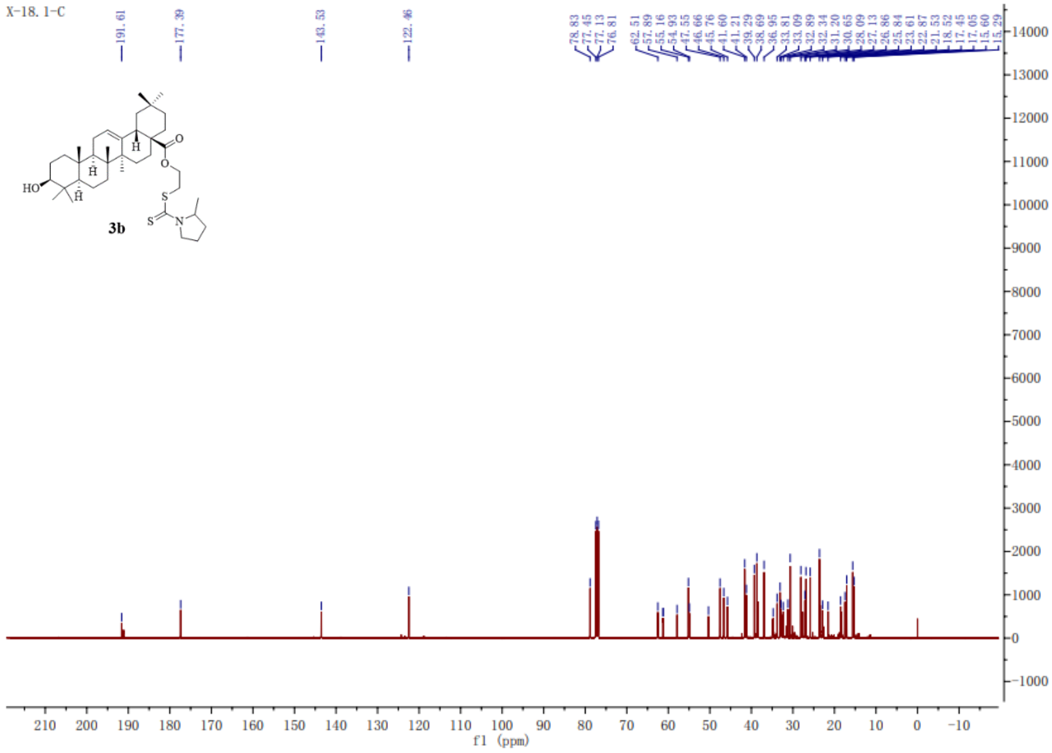

Figure S6. ^{13}C NMR spectrum of 3b.

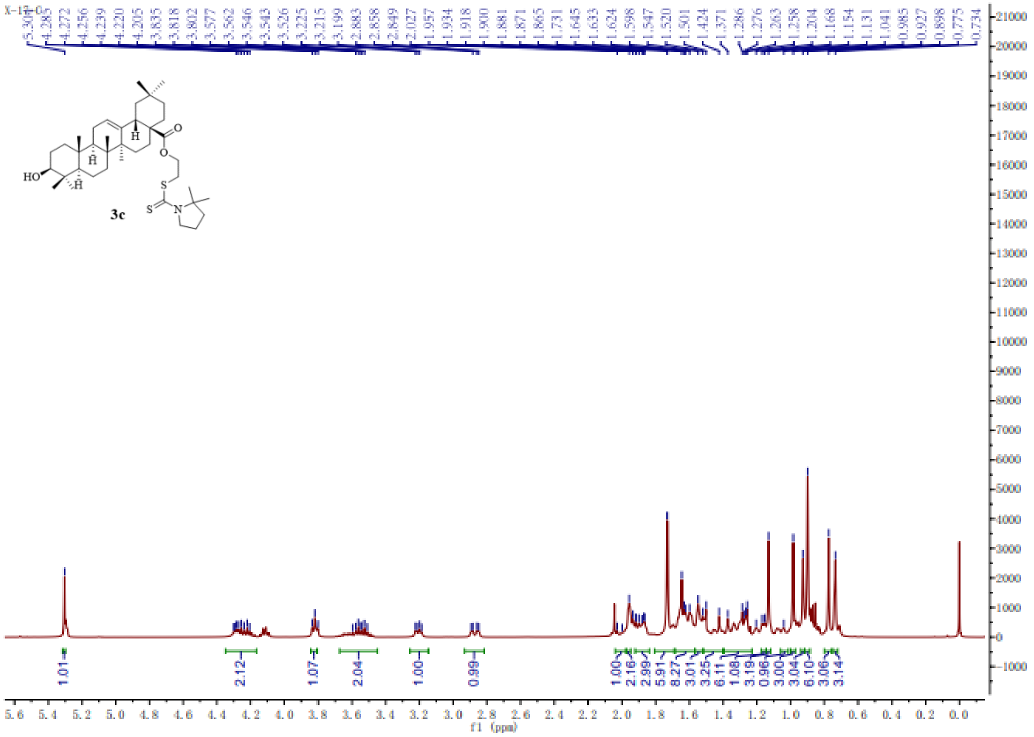

Figure S7. ^{1}**H NMR spectrum of 3c**.

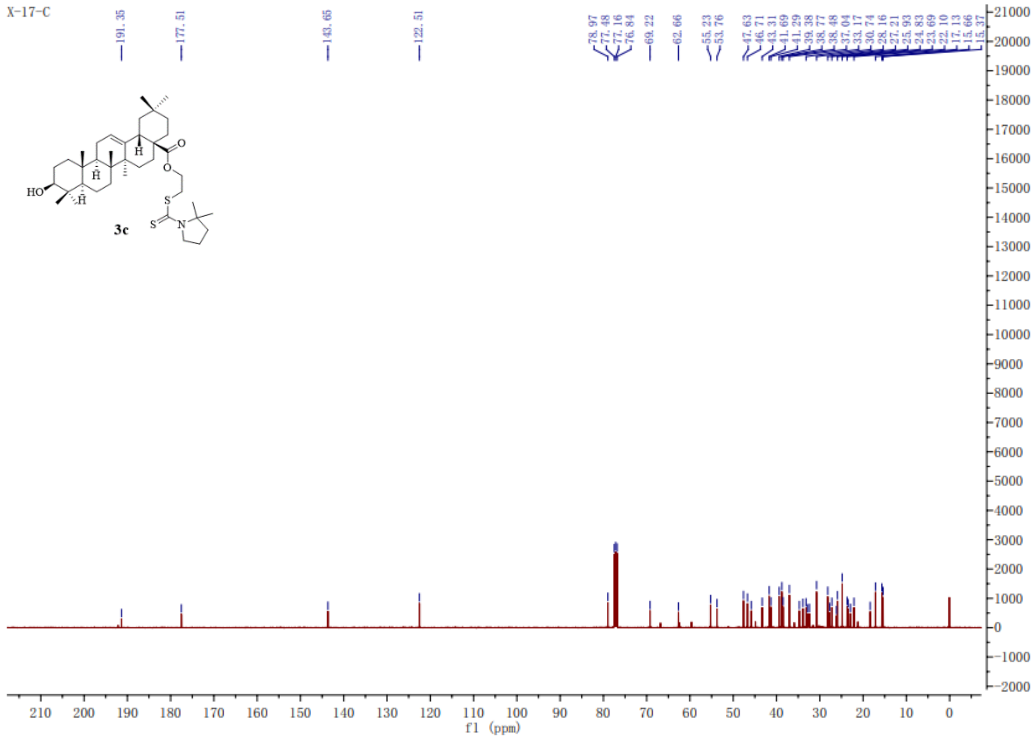

Figure S8. ^{13}**C NMR spectrum of 3c**.

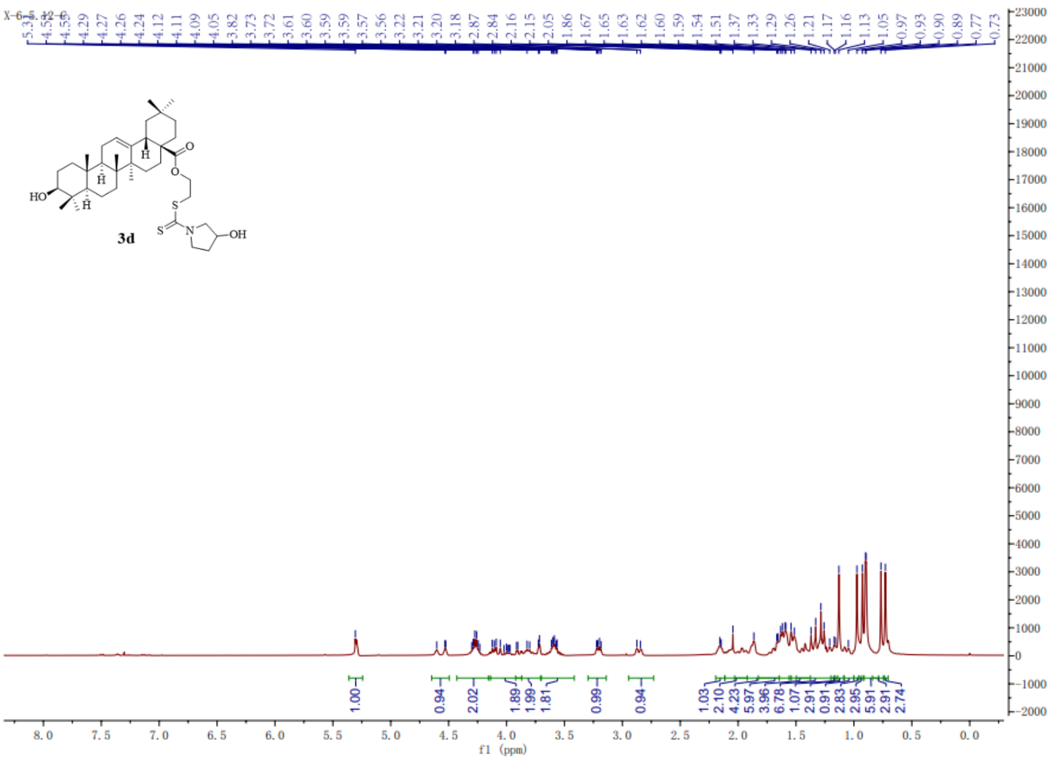

Figure S9. ^{1}H NMR spectrum of 3d.

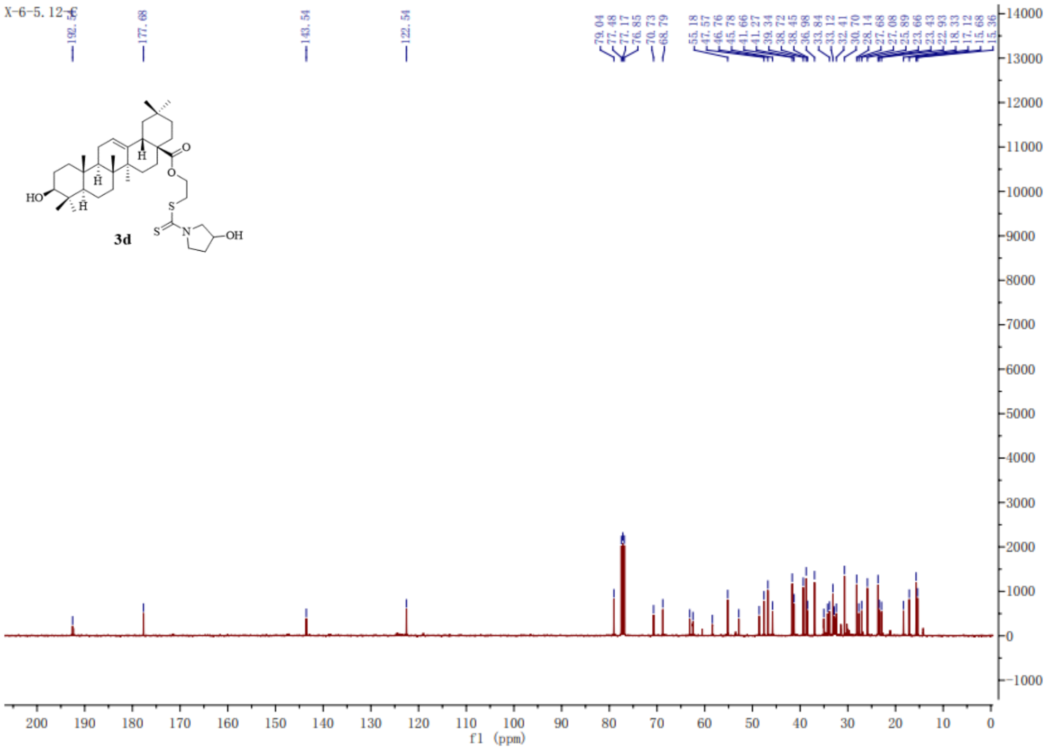

Figure S10. ^{13}C NMR spectrum of 3d.

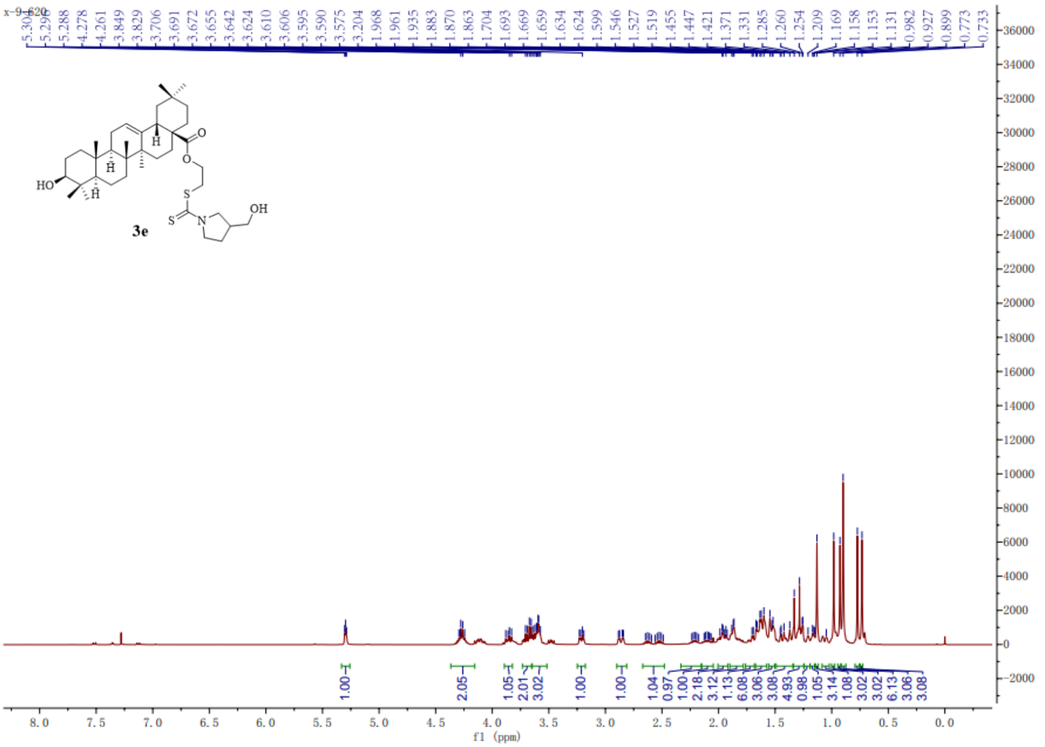

Figure S11. ^{1}H NMR spectrum of 3e.

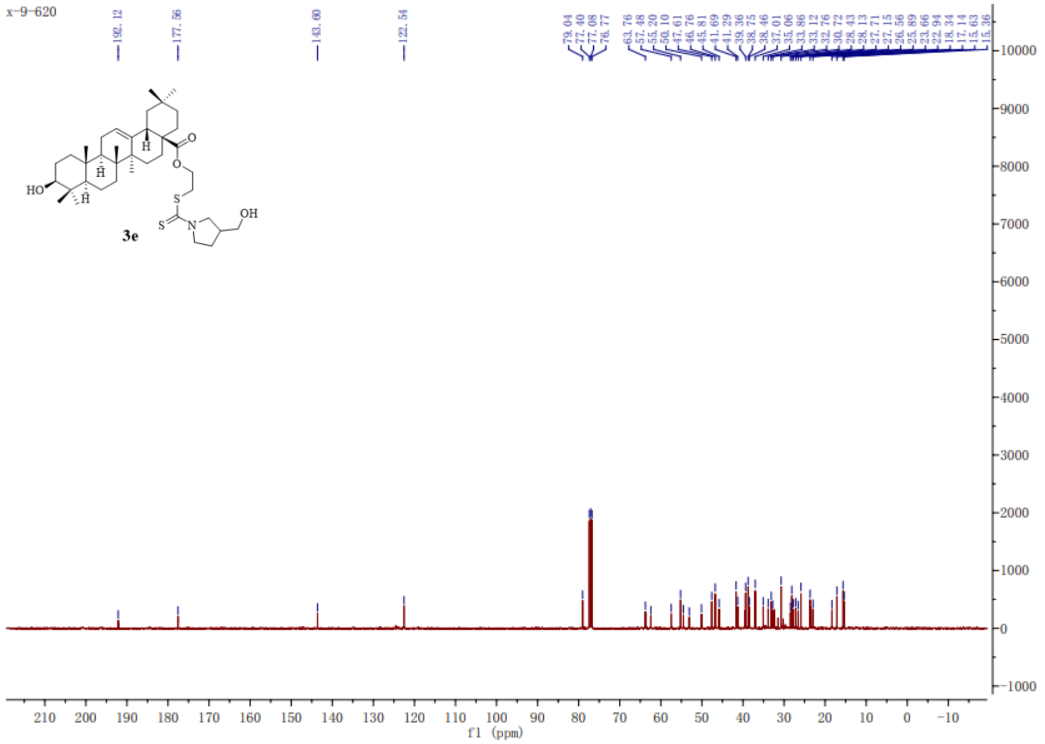

Figure S12. ^{13}C NMR spectrum of 3e.

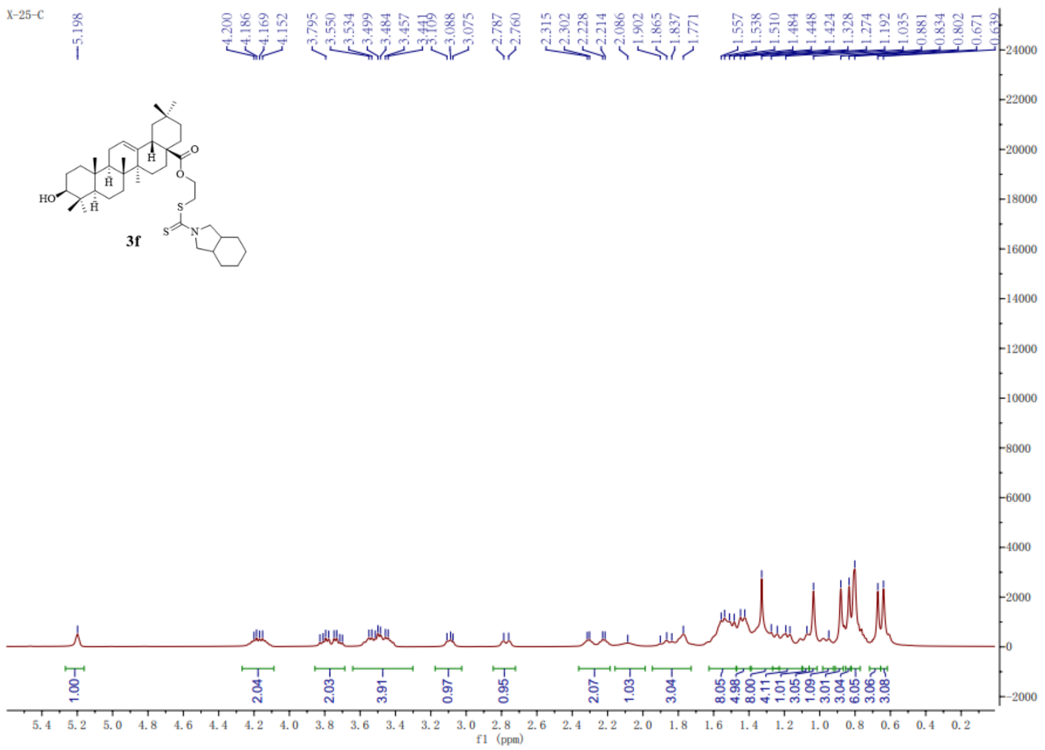

Figure S13. ^{1}H NMR spectrum of 3f.

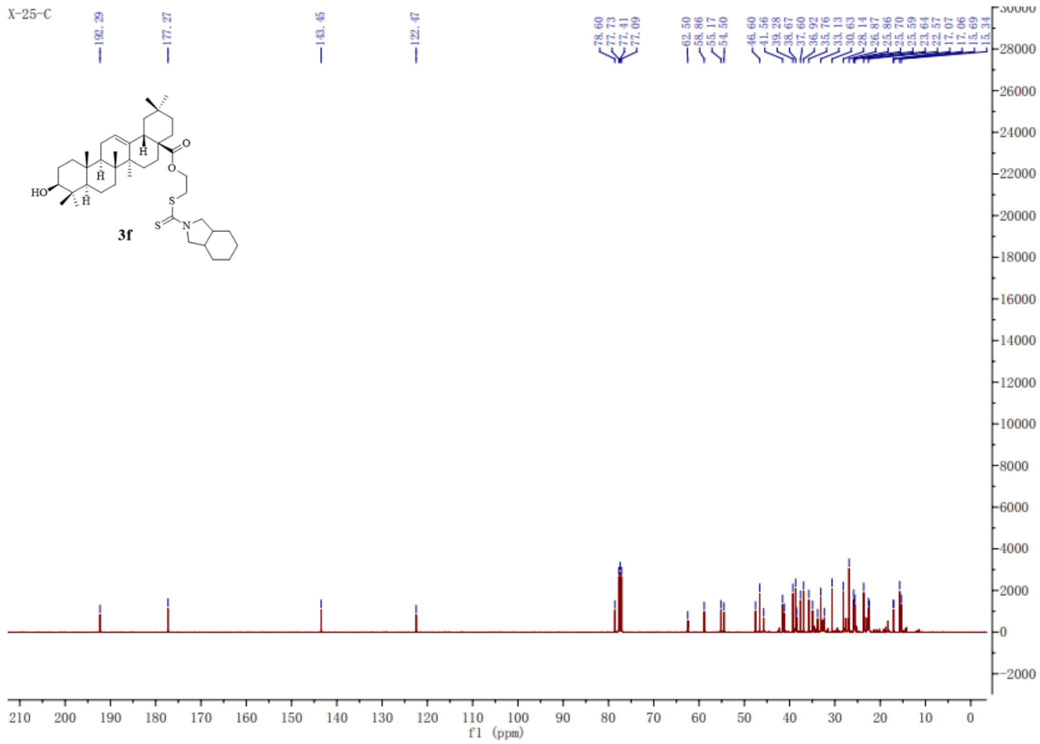

Figure S14. ^{13}C NMR spectrum of 3f.

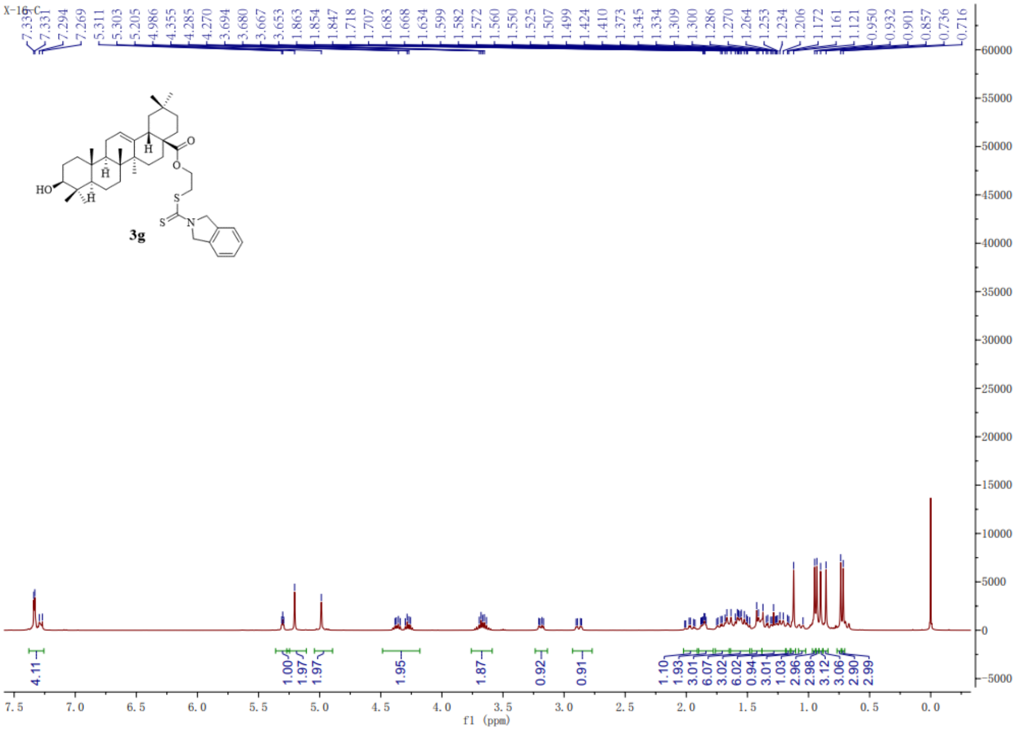

Figure S15. ^{1}H NMR spectrum of 3g.

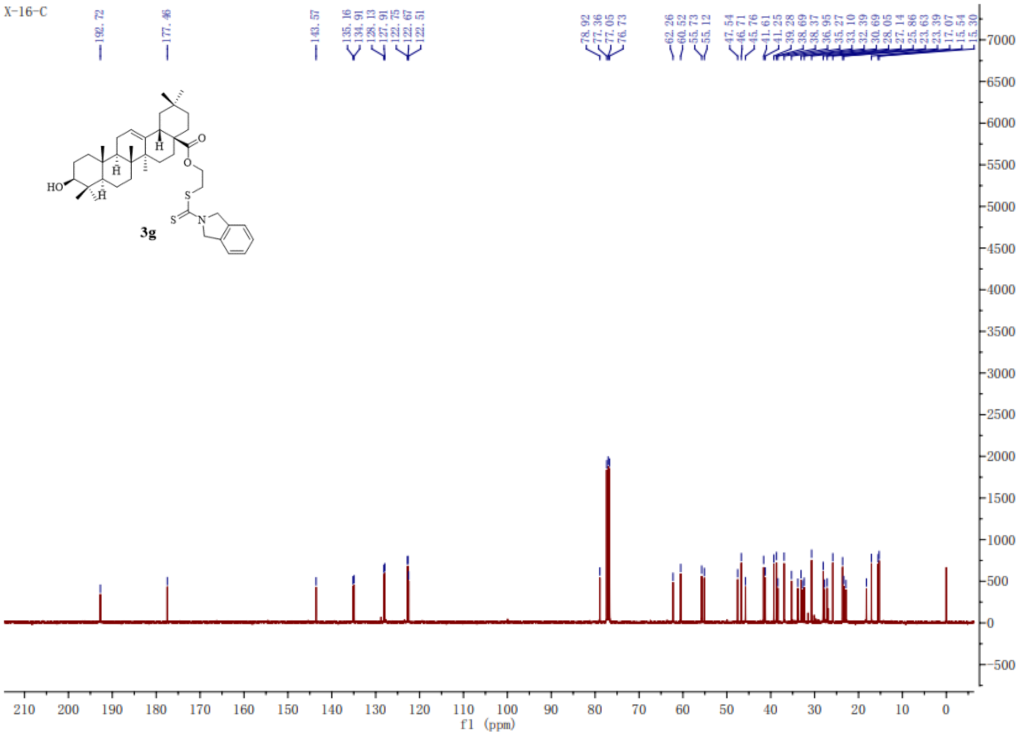

Figure S16. ^{13}C NMR spectrum of 3g.

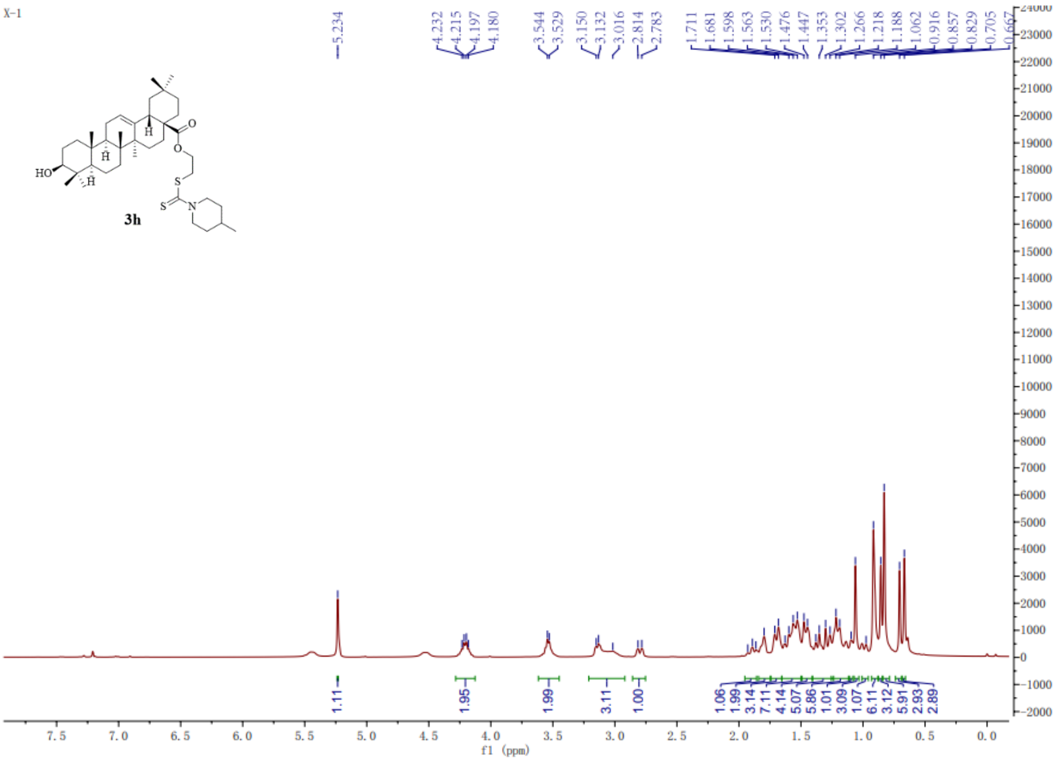

Figure S17. ^{1}H NMR spectrum of 3h.

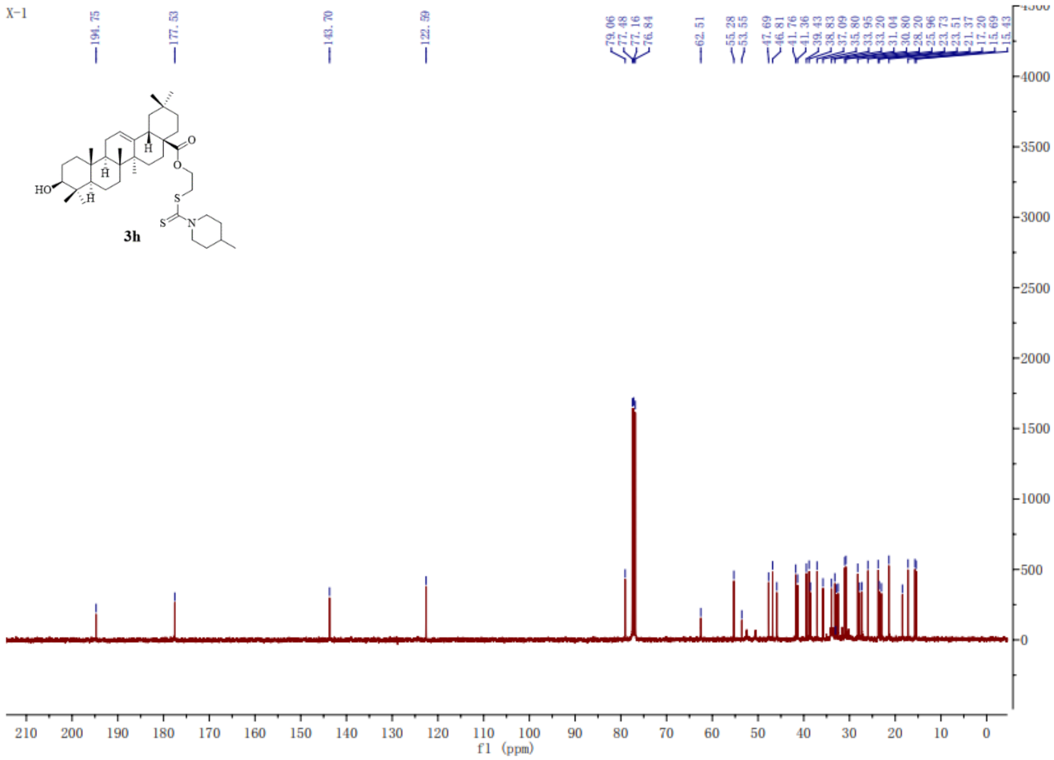

Figure S18. ^{13}C NMR spectrum of 3h.

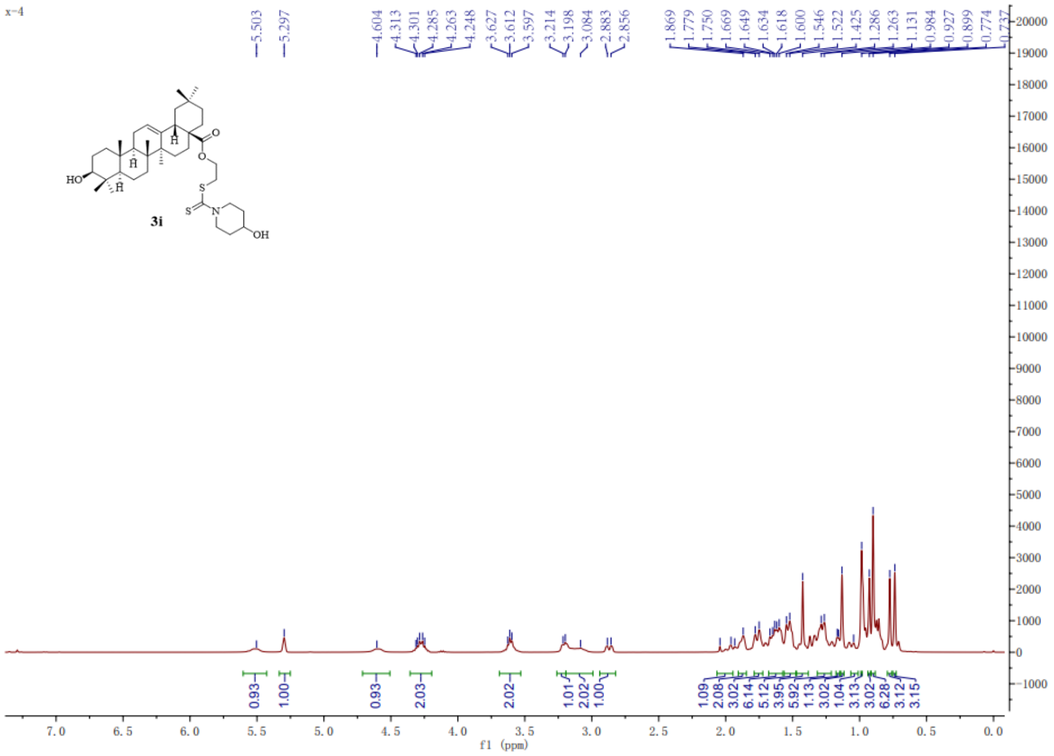

Figure S19. ^{1}H NMR spectrum of 3i.

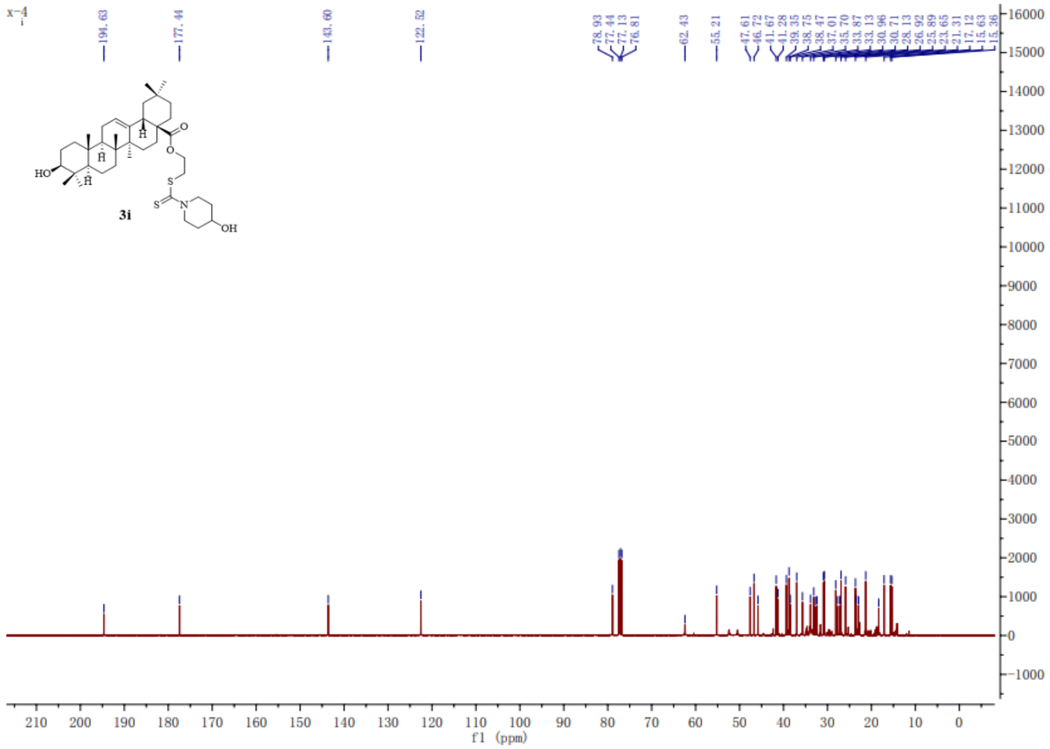

Figure S20. ^{13}C NMR spectrum of 3i.

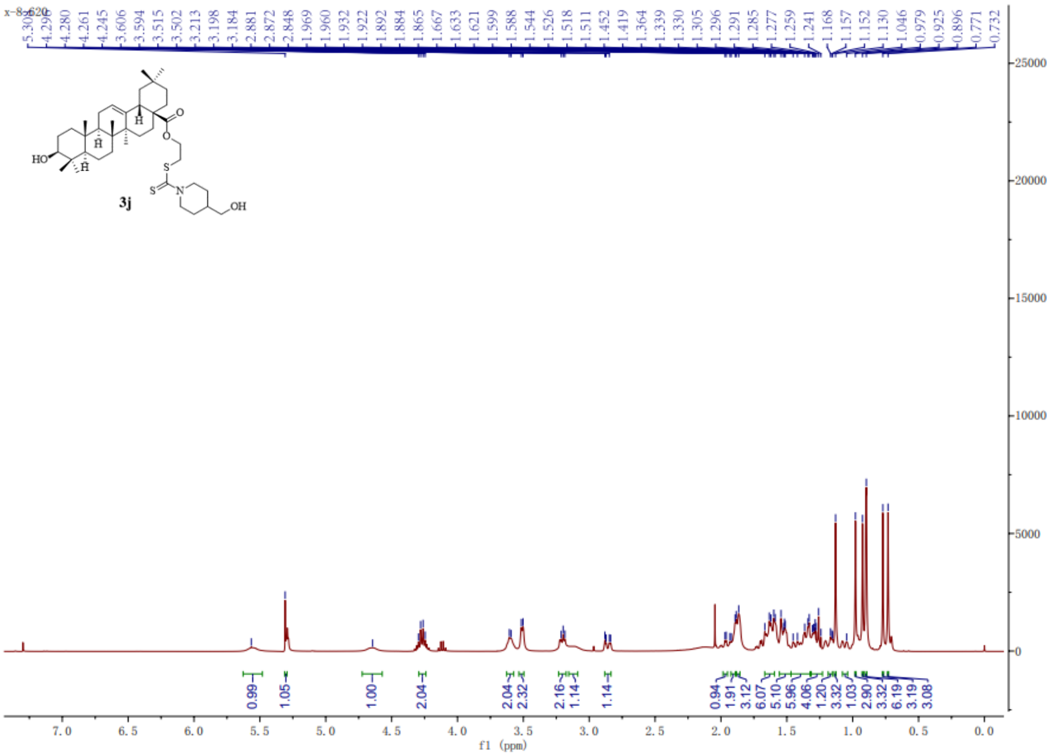

Figure S21. **^{1}H NMR spectrum of 3j**.

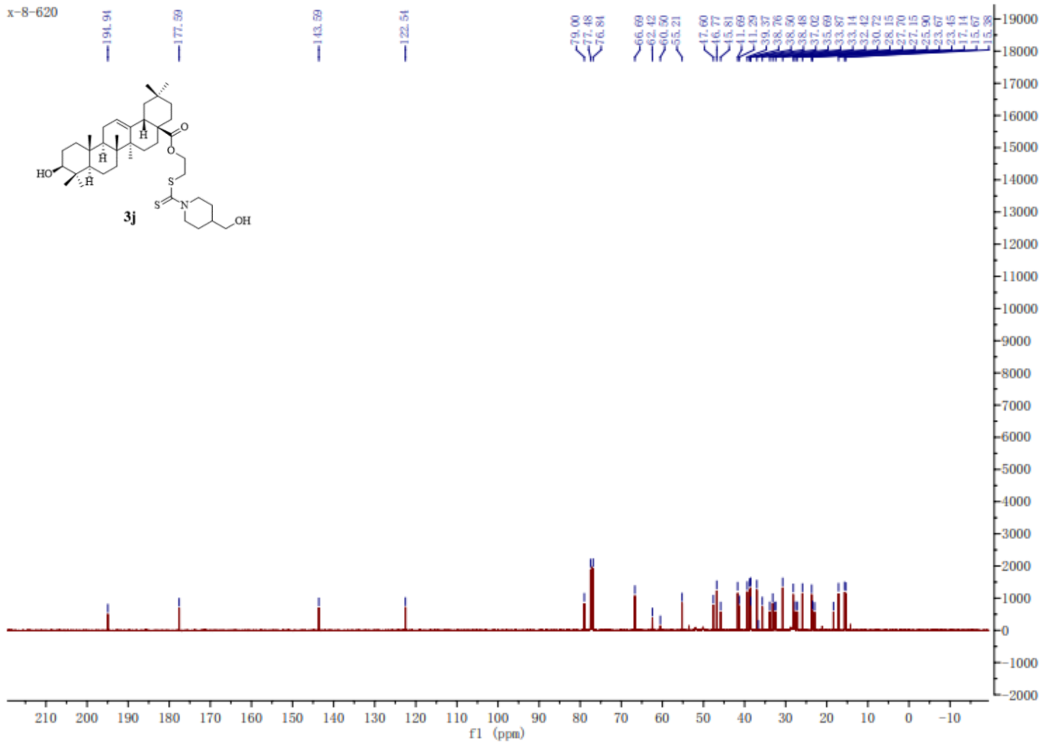

Figure S22. **^{13}C NMR spectrum of 3j**.

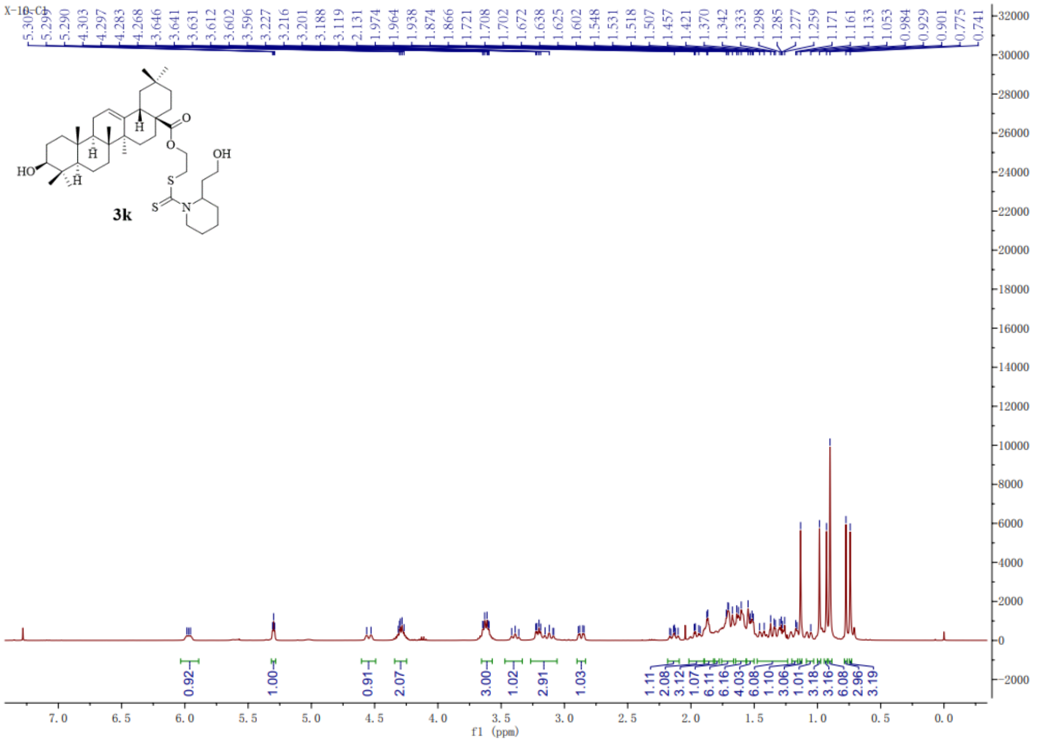

Figure S23. **^{1}H NMR spectrum of 3k**.

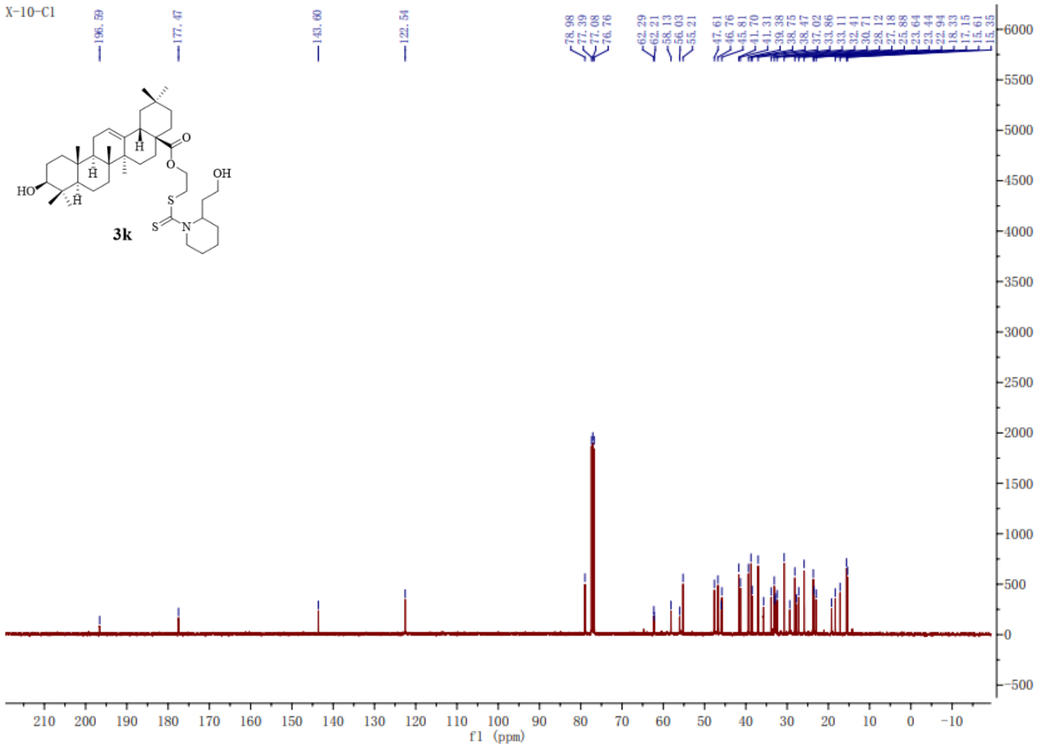

Figure S24. **^{13}C NMR spectrum of 3k**.

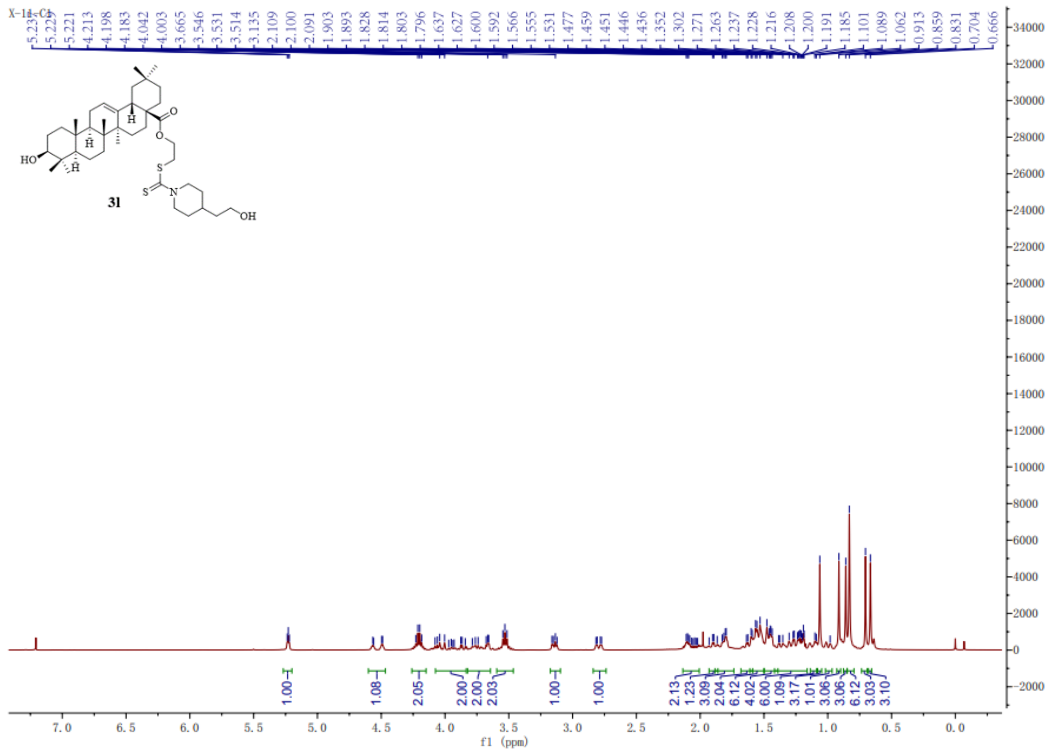

Figure S25. ^{1}H NMR spectrum of 3l.

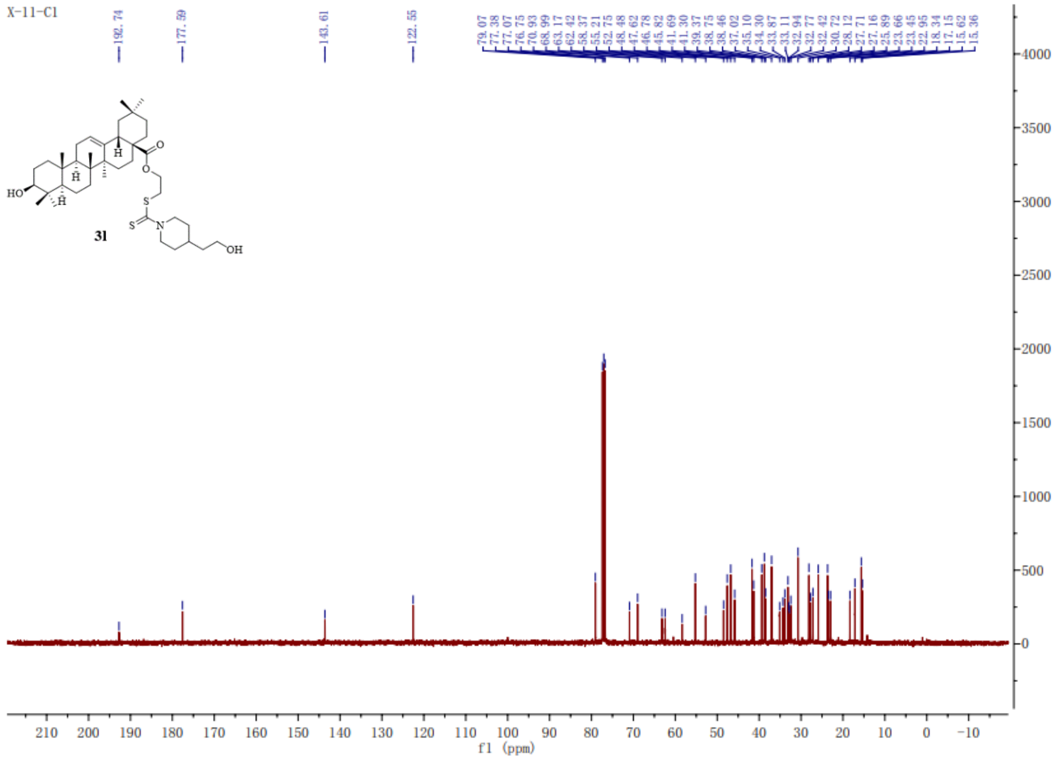

Figure S26. ^{13}C NMR spectrum of 3l.

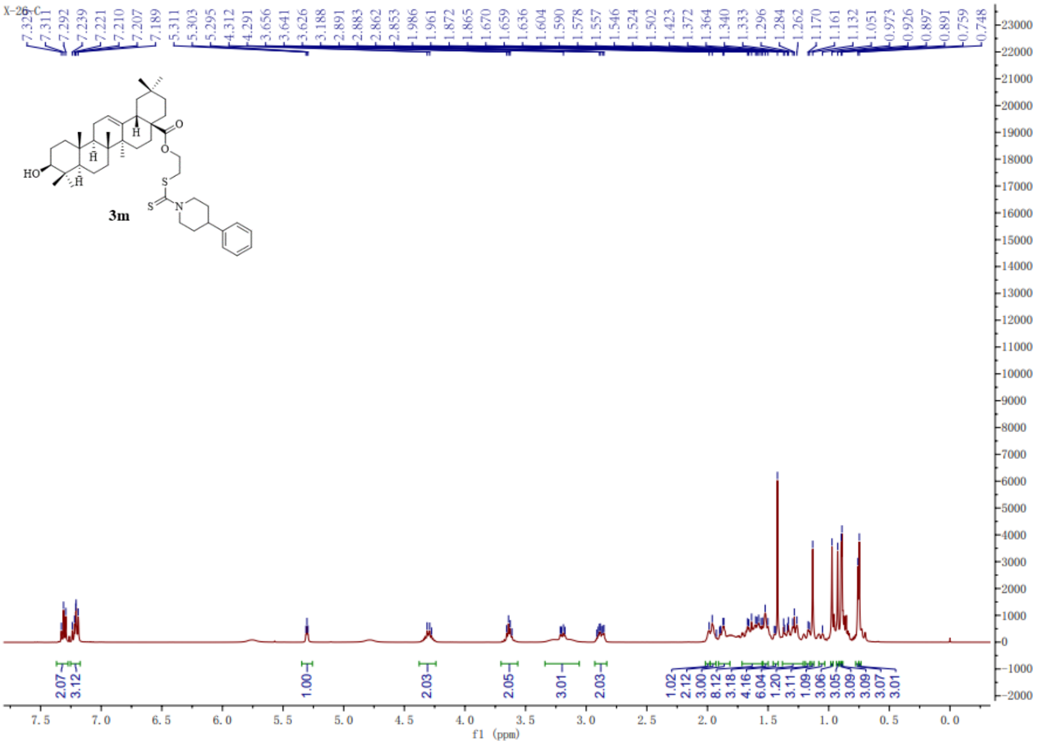

Figure S27. ^{1}H NMR spectrum of 3m.

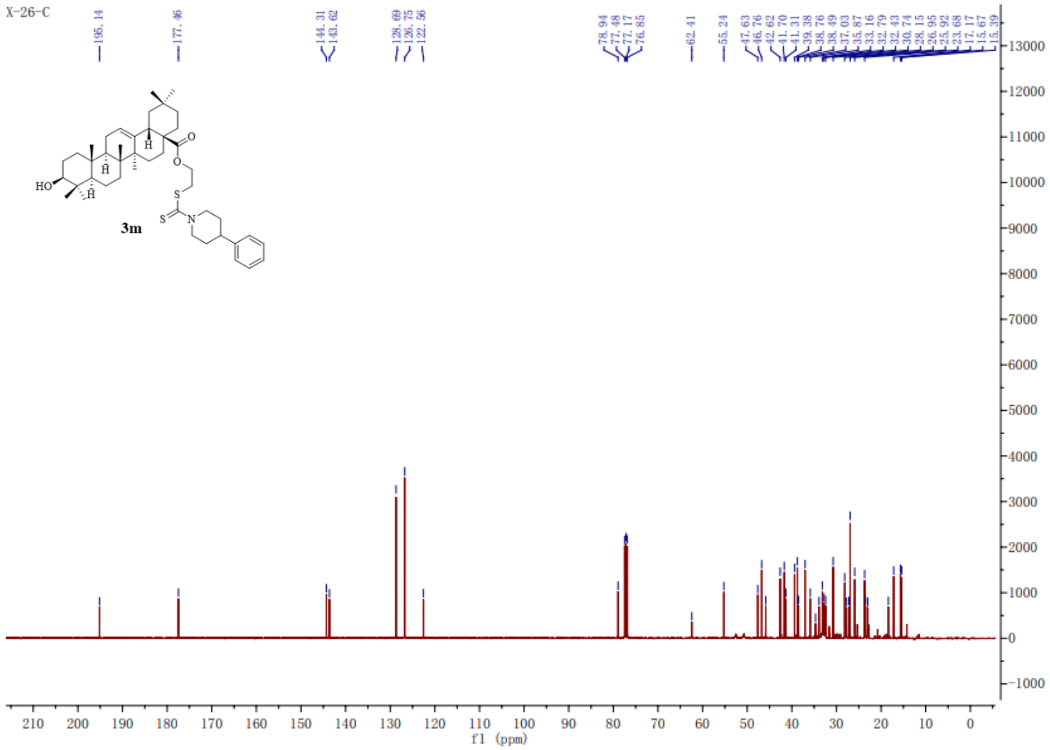

Figure S28. ^{13}C NMR spectrum of 3m.

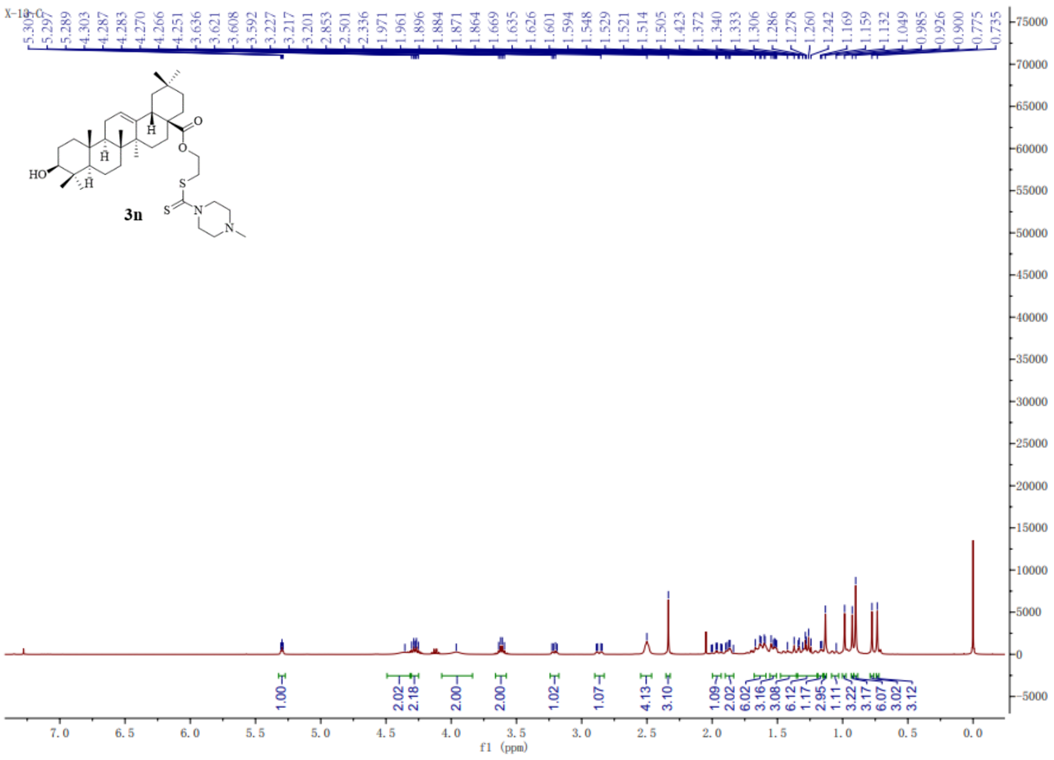

Figure S29. ^{1}**H NMR spectrum of 3n**.

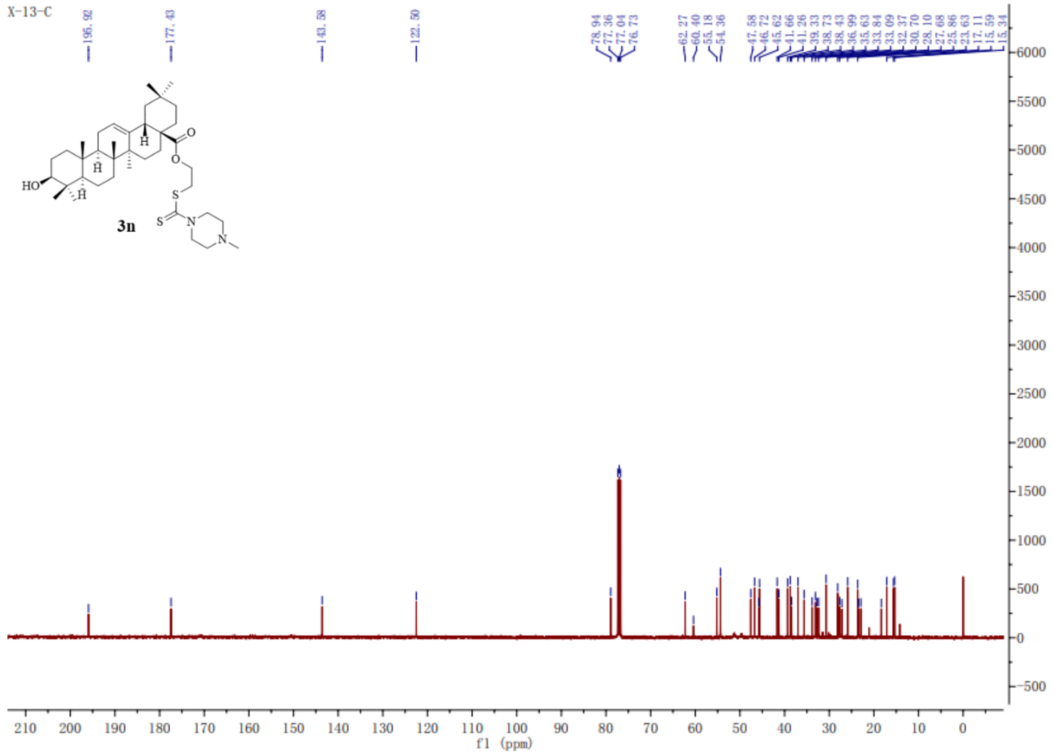

Figure S30. ^{13}**C NMR spectrum of 3n**.

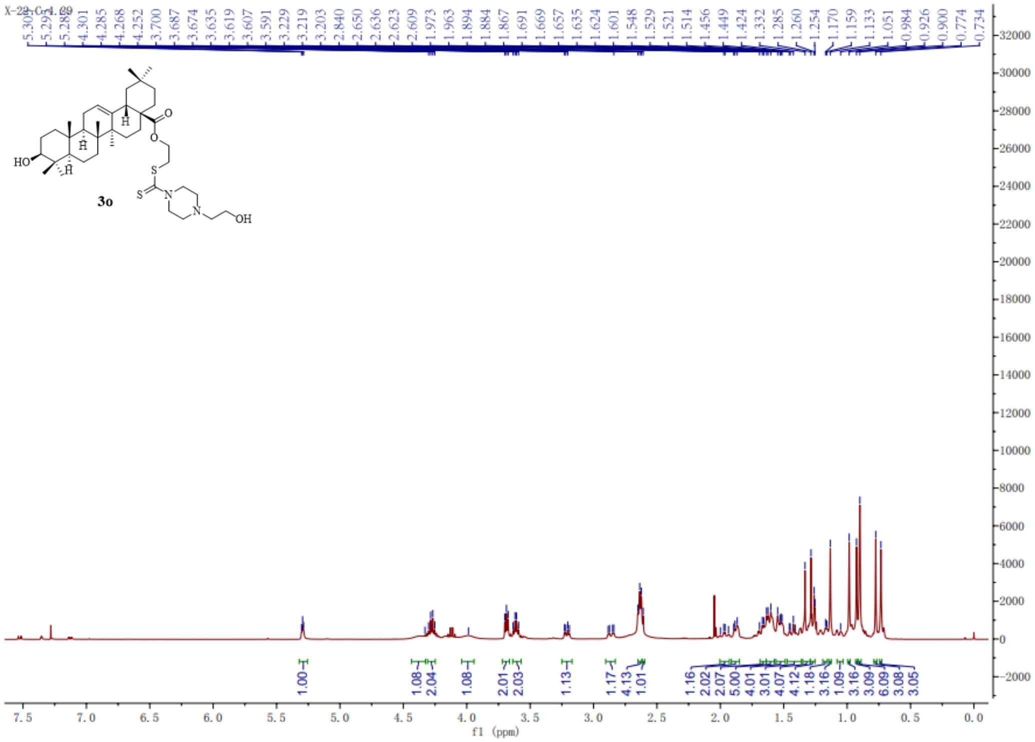

Figure S31. ^{1}H NMR spectrum of 3o.

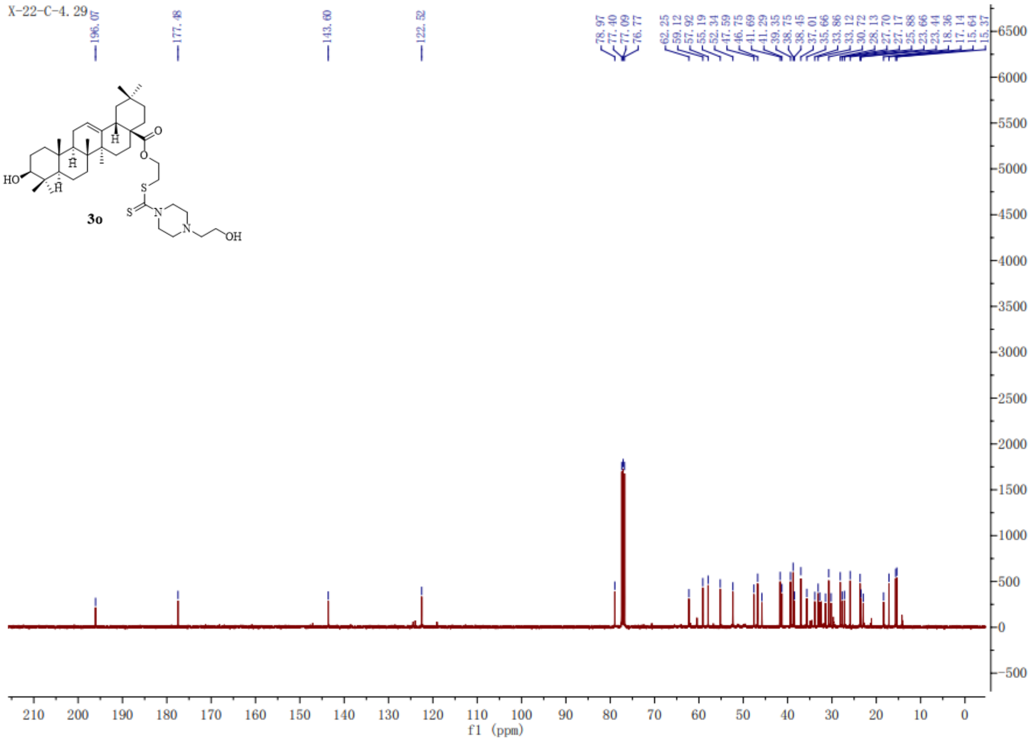

Figure S32. ^{13}C NMR spectrum of 3o.

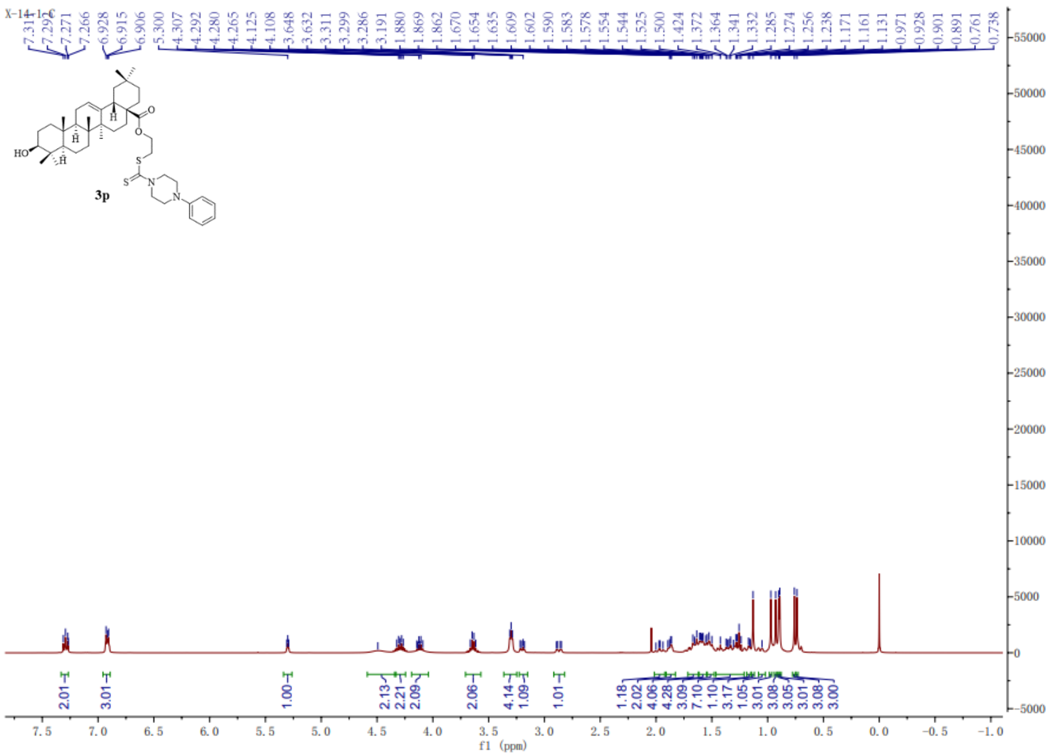

Figure S33. ^{1}H NMR spectrum of 3p.

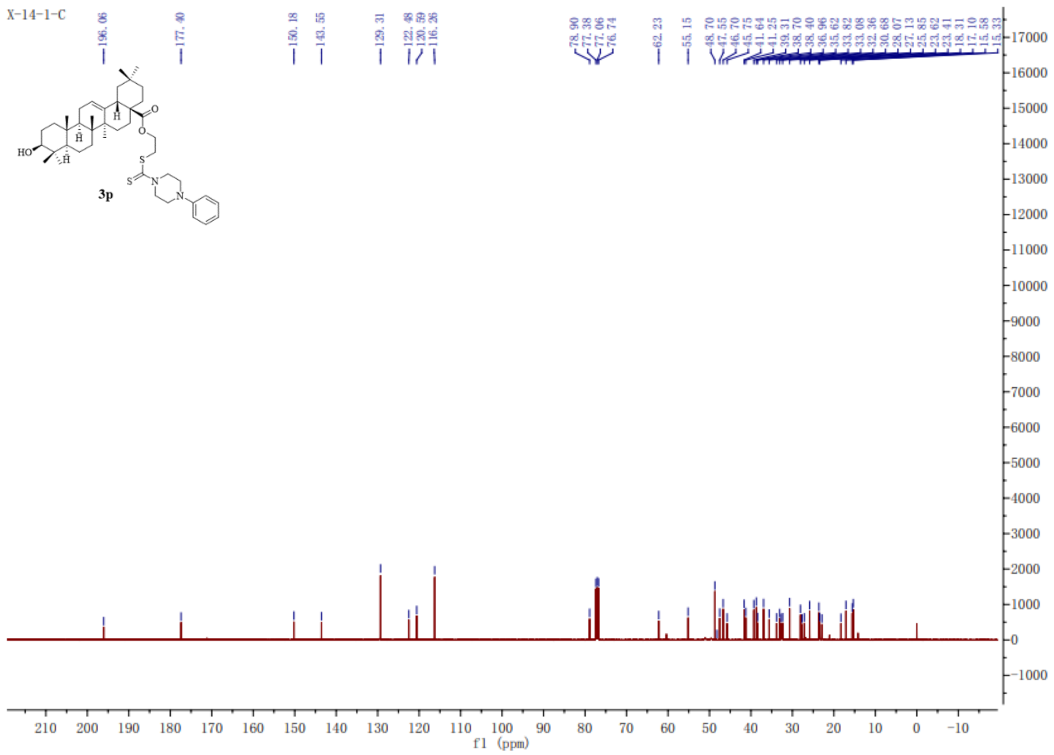

Figure S34. ^{13}C NMR spectrum of 3p.

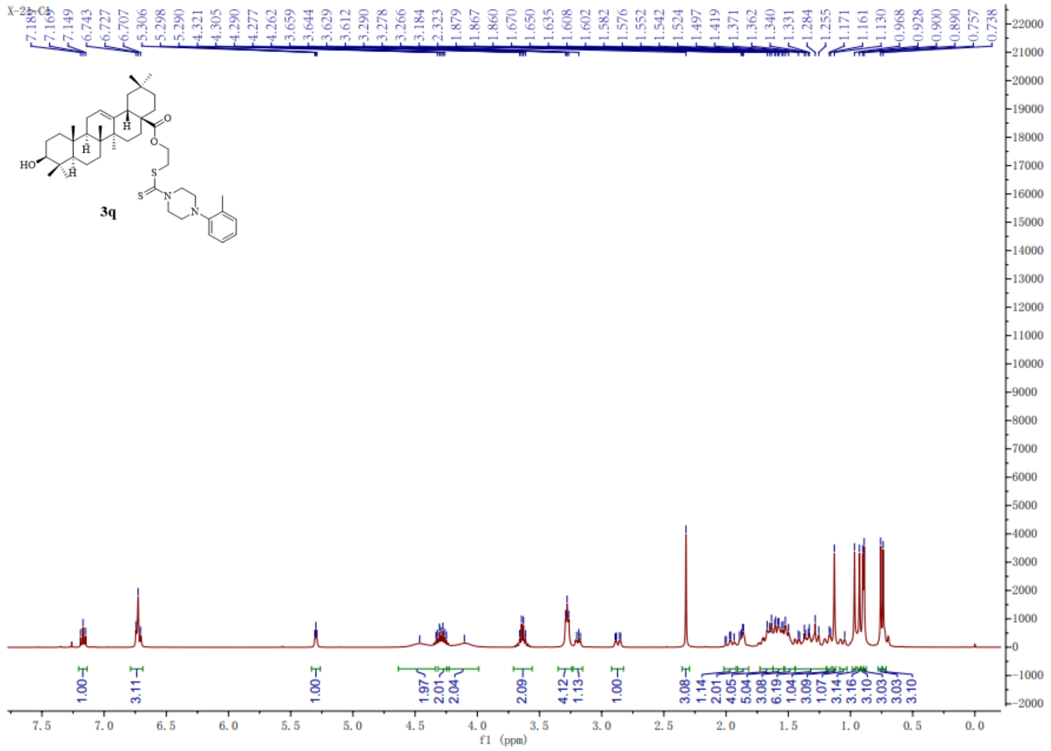

Figure S35. ^{1}**H NMR spectrum of 3q**.

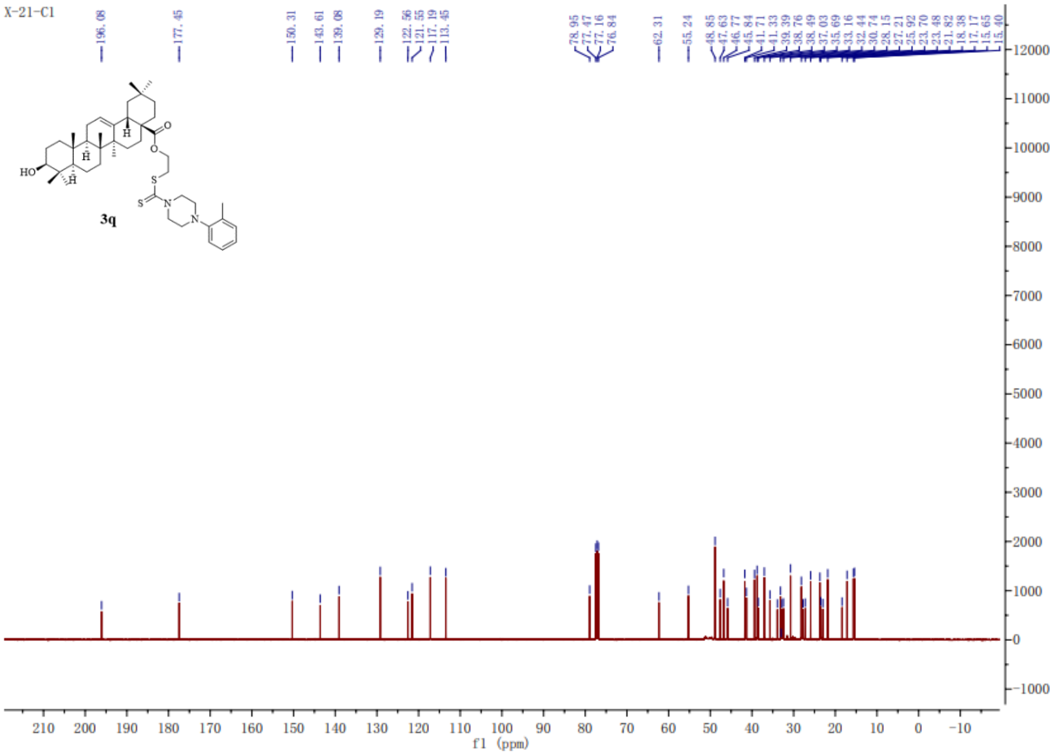

Figure S36. ^{13}**C NMR spectrum of 3q**.

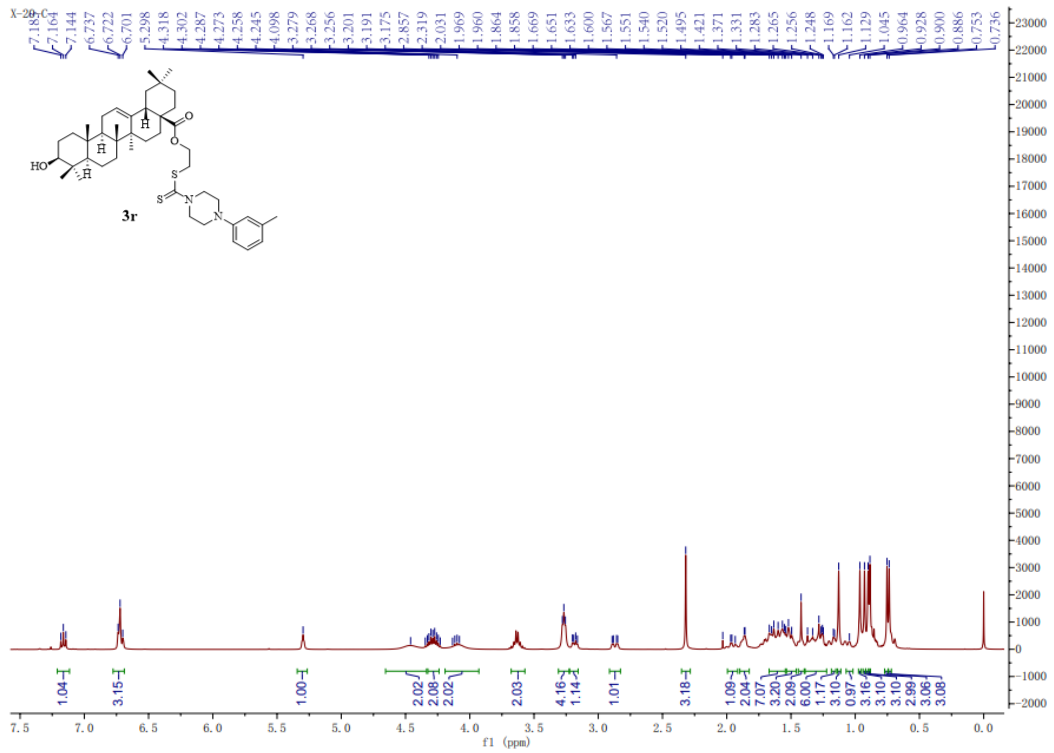

Figure S37. ^{1}**H NMR spectrum of 3r**.

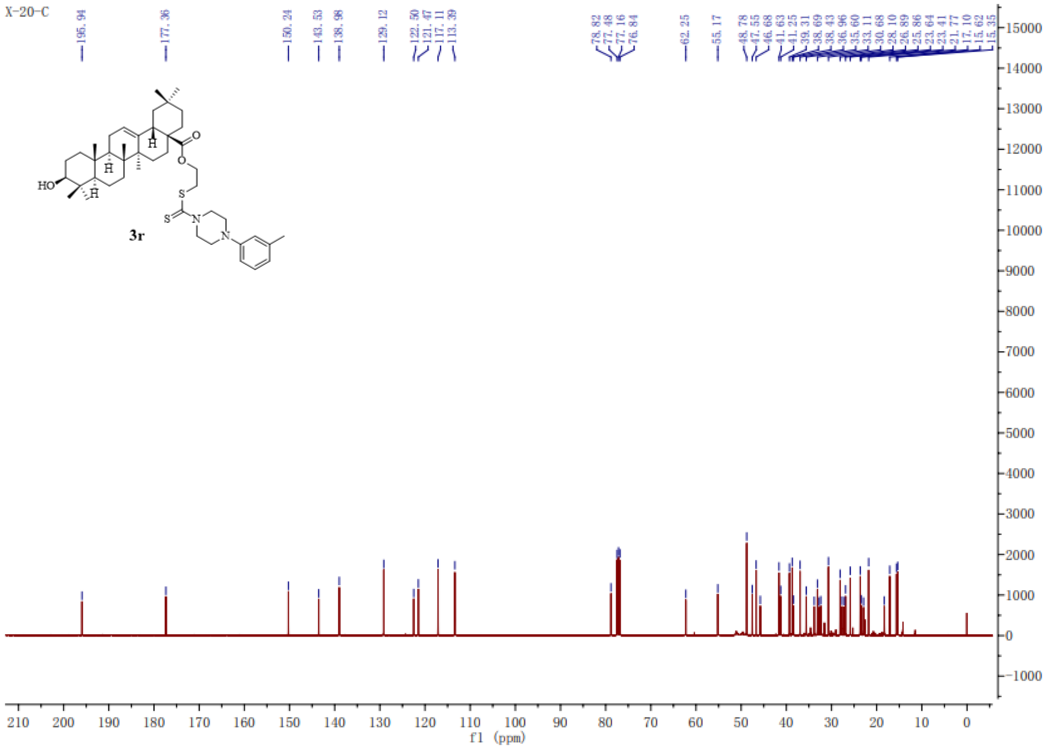

Figure S38. ^{13}**C NMR spectrum of 3r**.

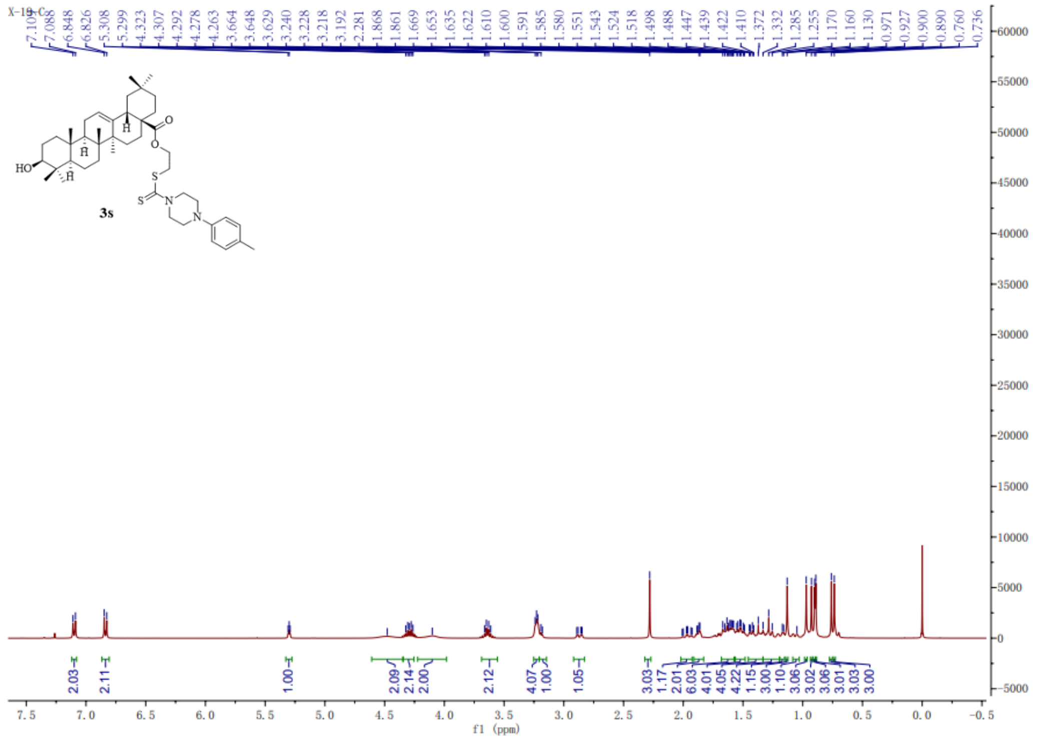

Figure S39. ^{1}H NMR spectrum of 3s.

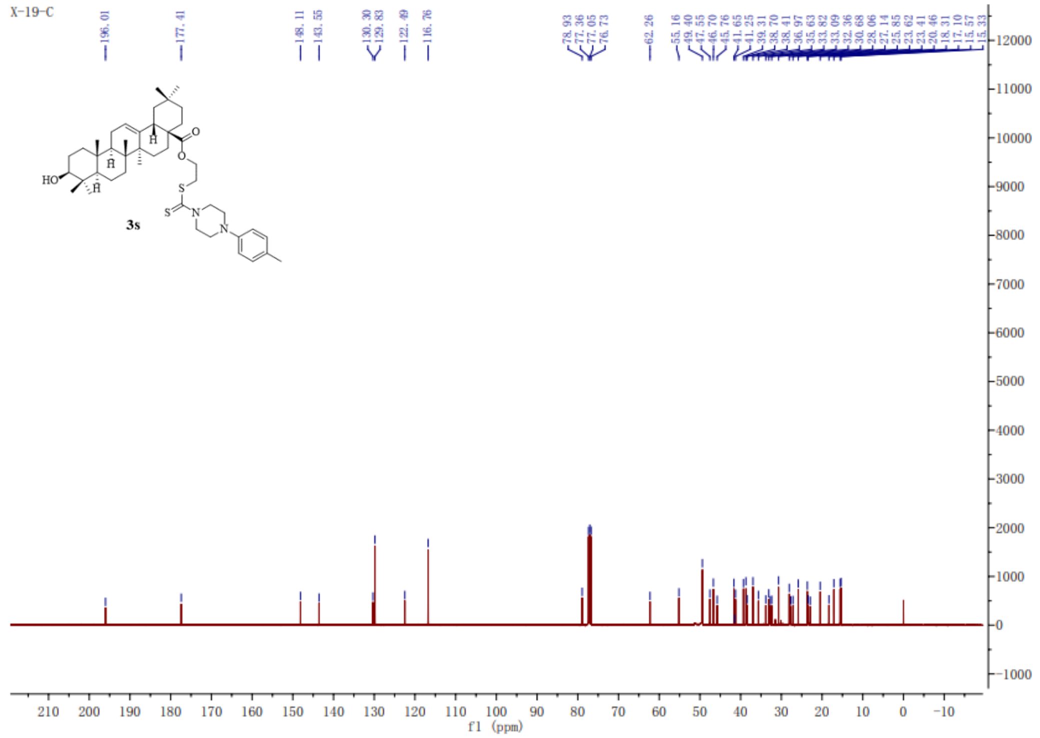

Figure S40. ^{13}C NMR spectrum of 3s.

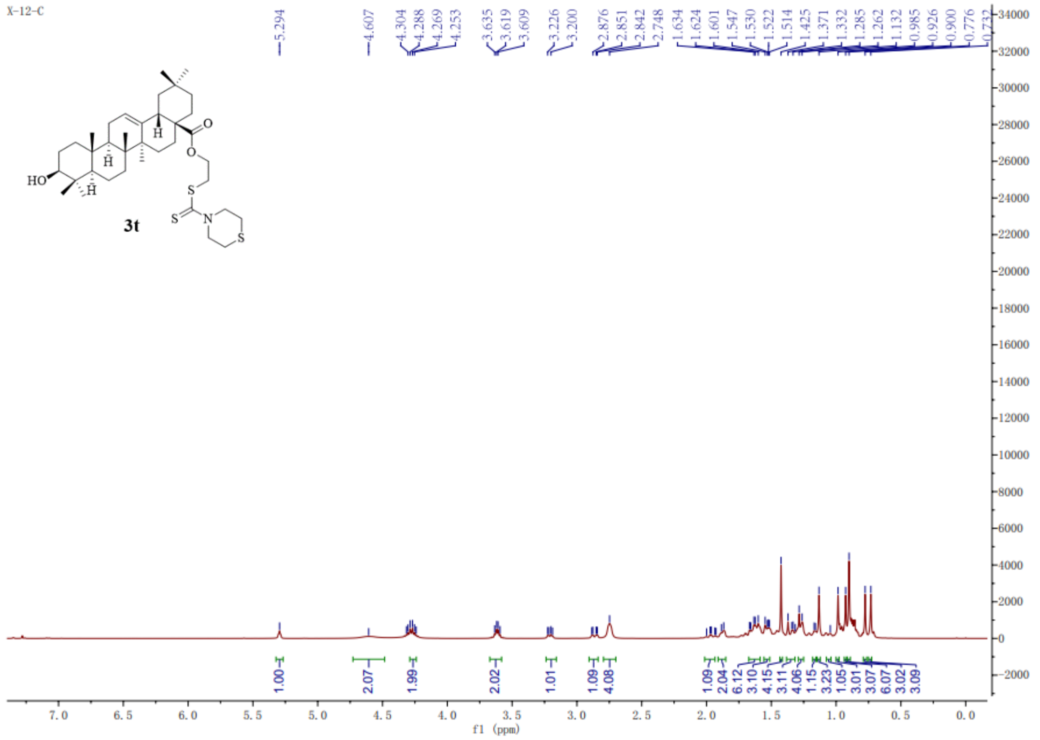

Figure S41. ^{1}H NMR spectrum of 3t.

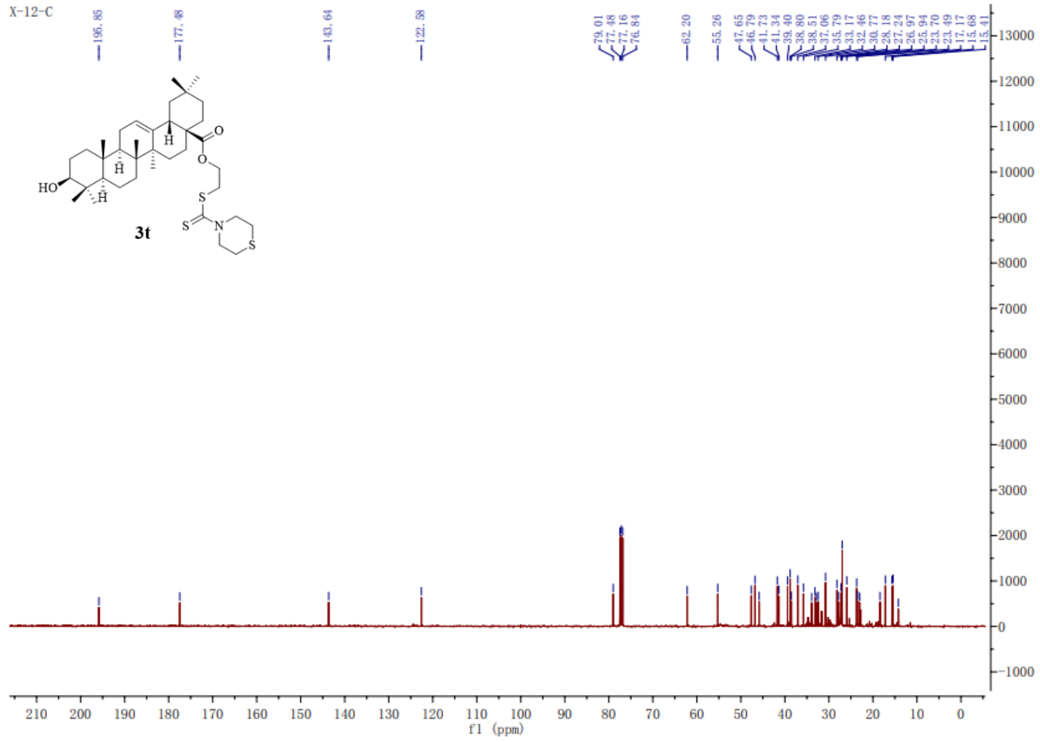

Figure S42. ^{13}C NMR spectrum of 3t.

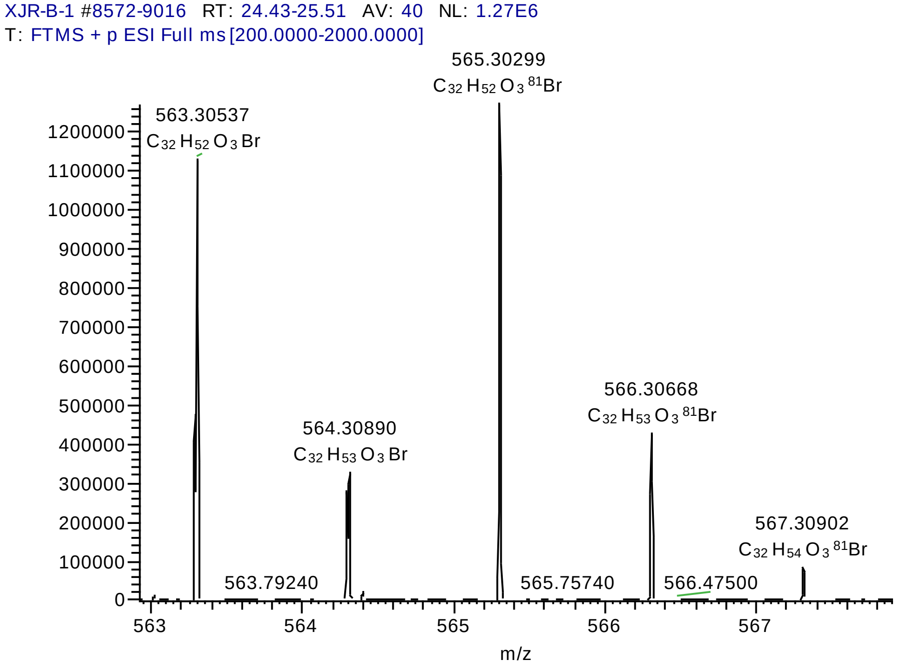

Figure S43. HRMS spectrum of 2.

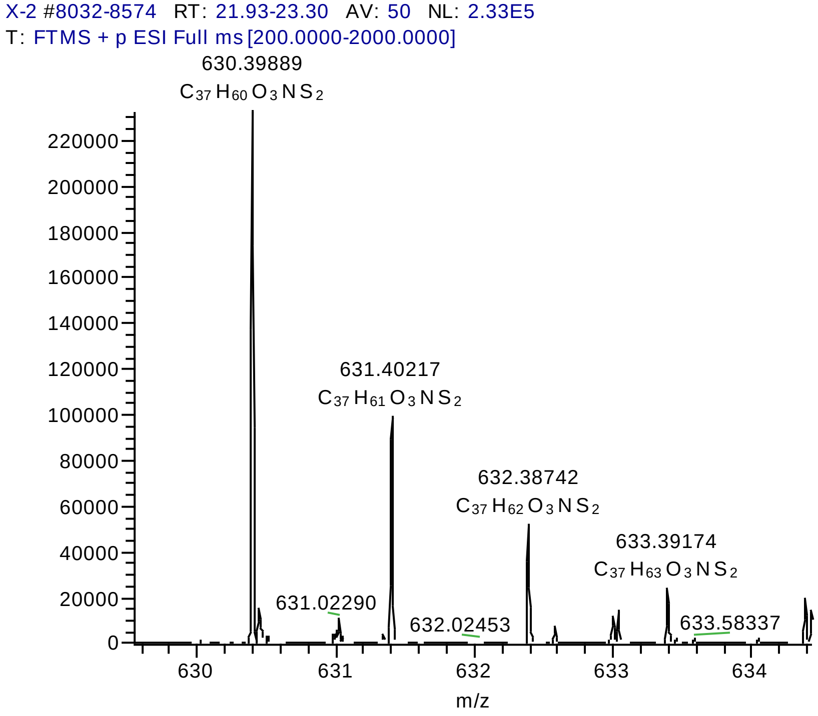

Figure S44. HRMS spectrum of 3a.

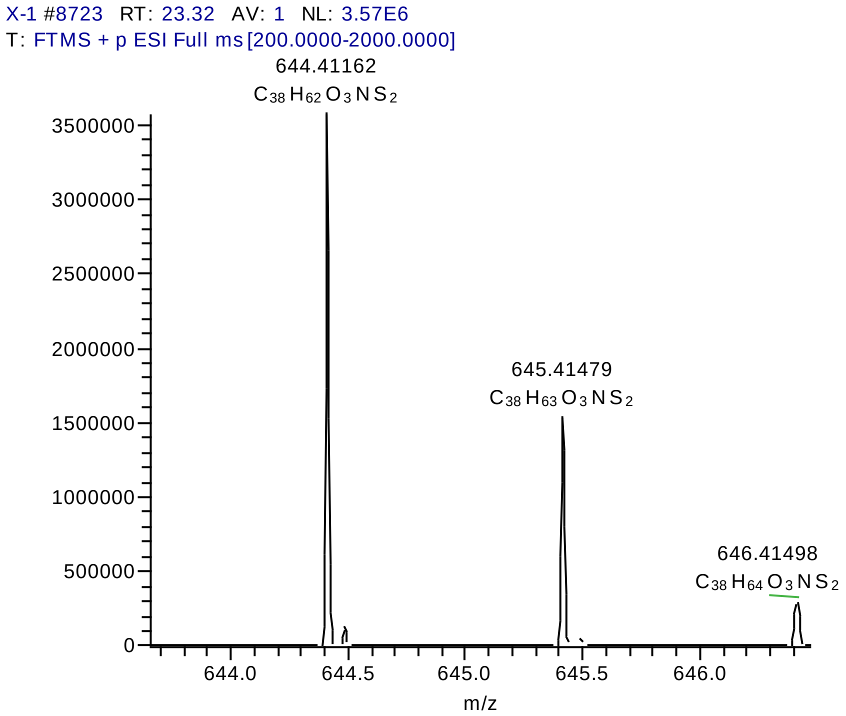

Figure S45. HRMS spectrum of 3b.

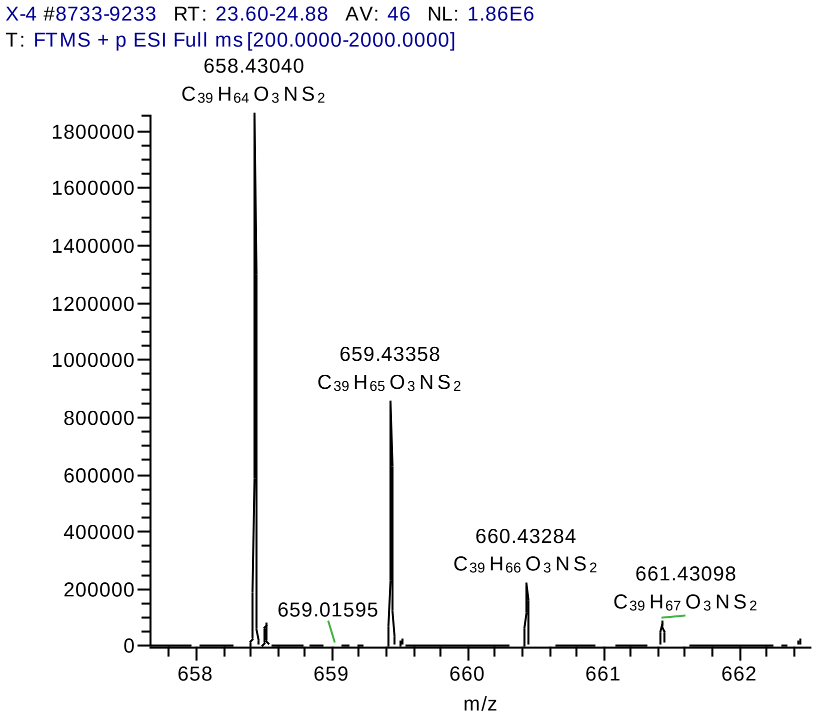

Figure S46. HRMS spectrum of 3c.

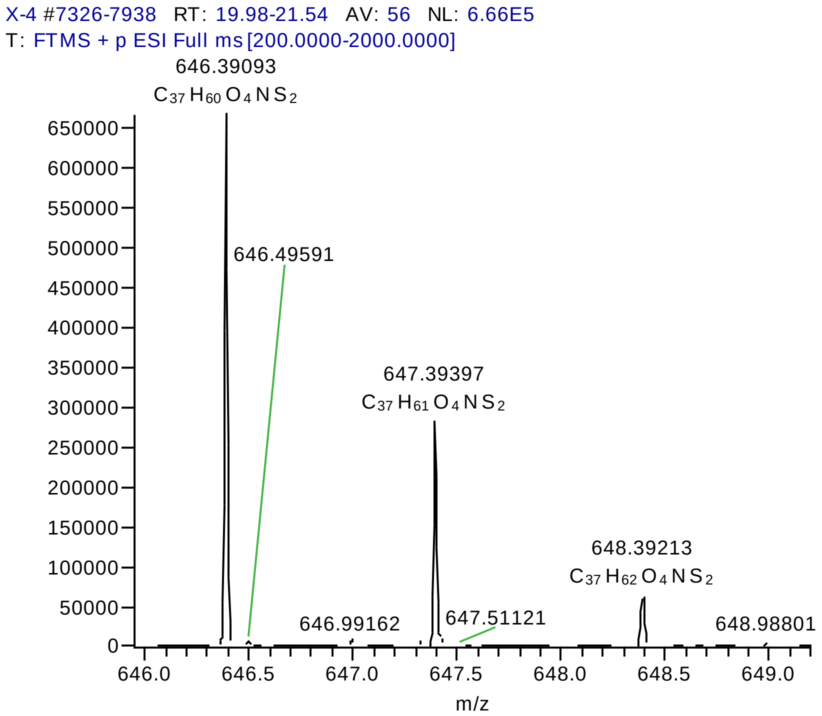

Figure S47. HRMS spectrum of 3d.

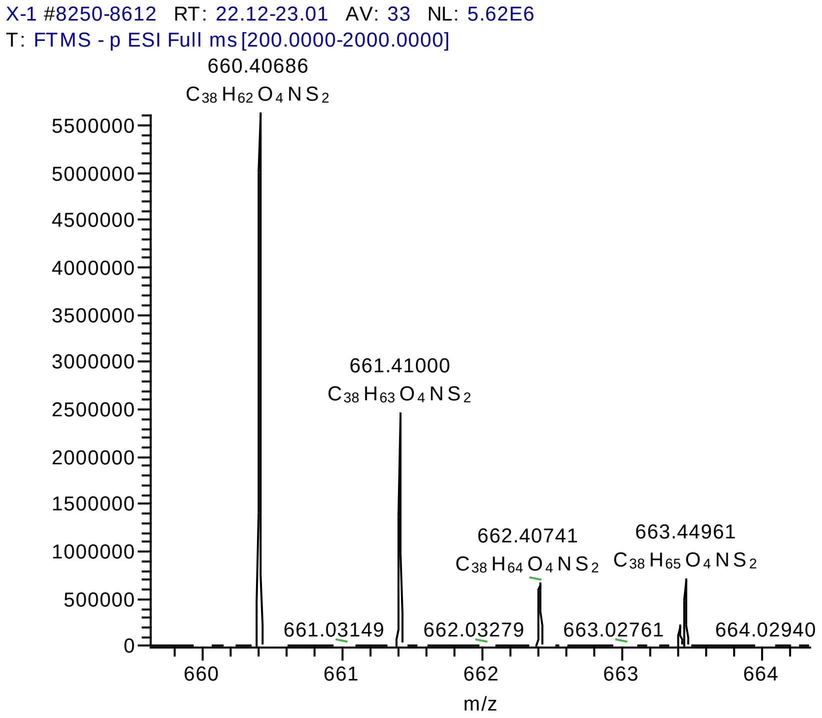

Figure S48. HRMS spectrum of 3e.

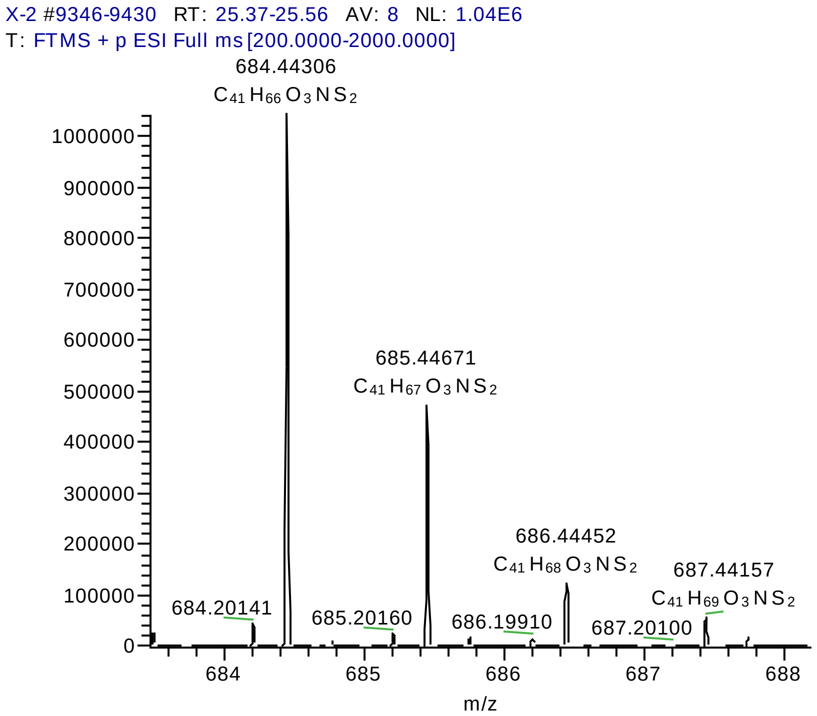

Figure S49. HRMS spectrum of 3f.

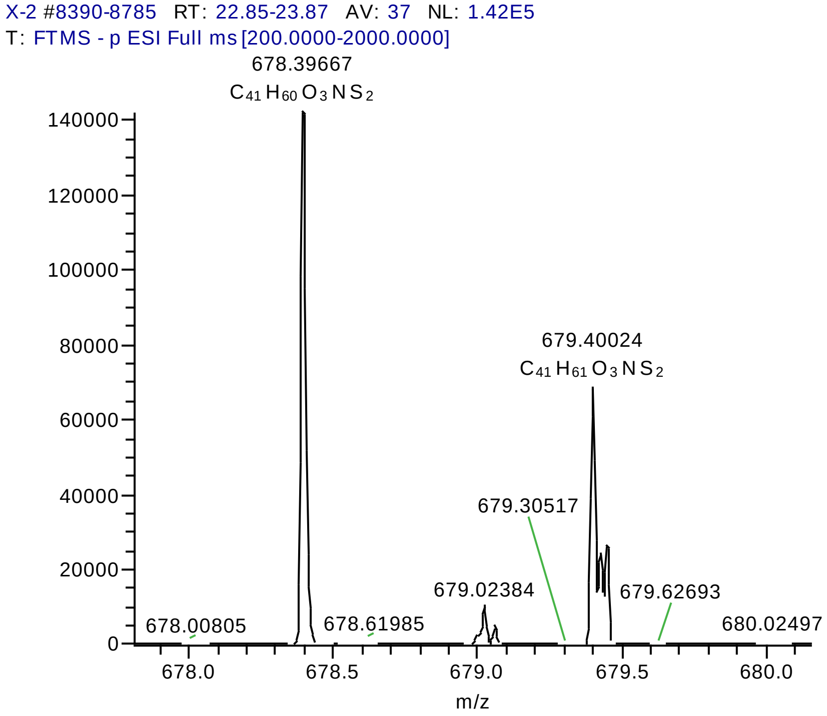

Figure S50. HRMS spectrum of 3g.

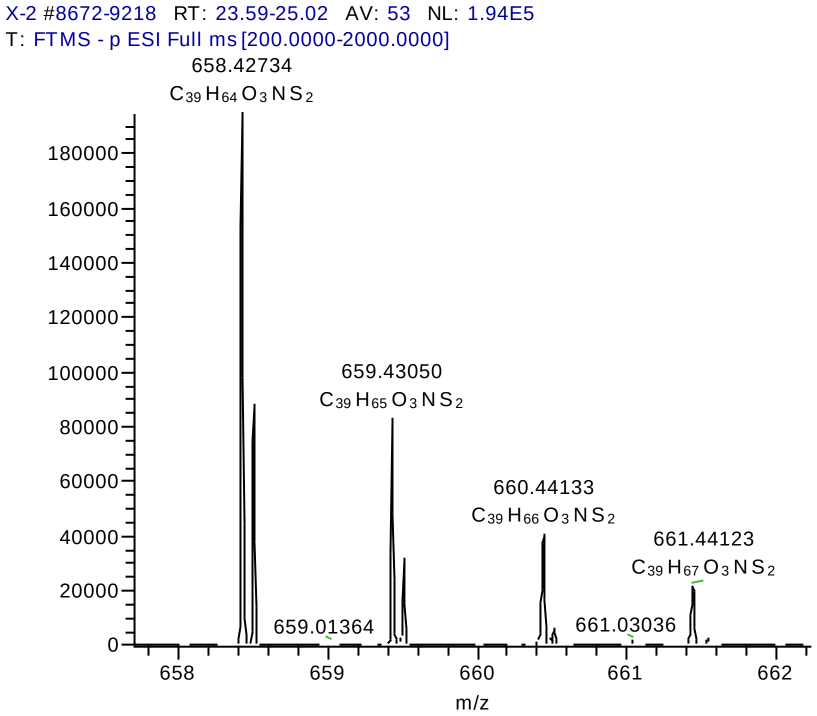

Figure S51. HRMS spectrum of 3h.

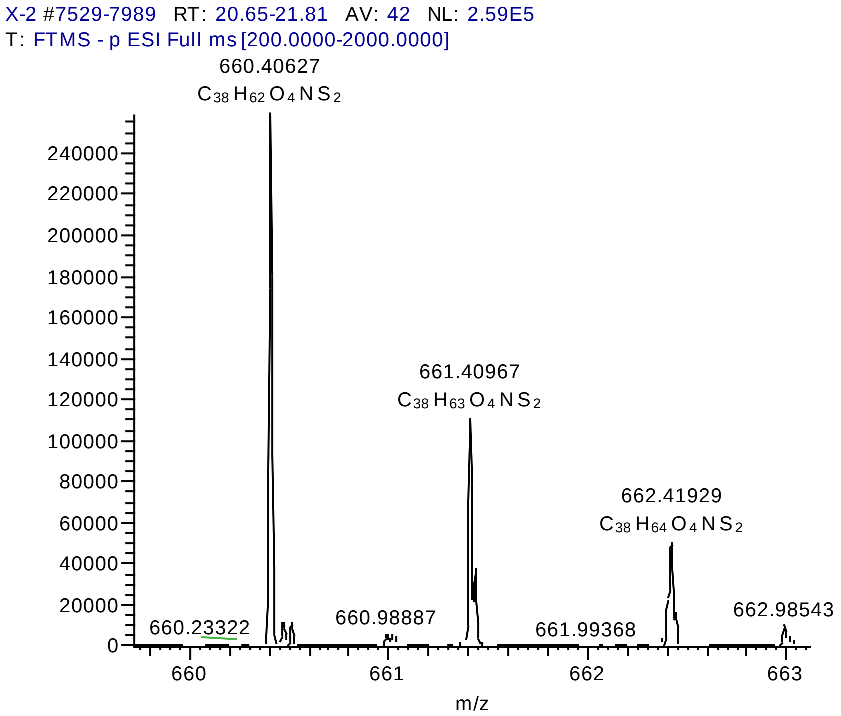

Figure S52. HRMS spectrum of 3i.

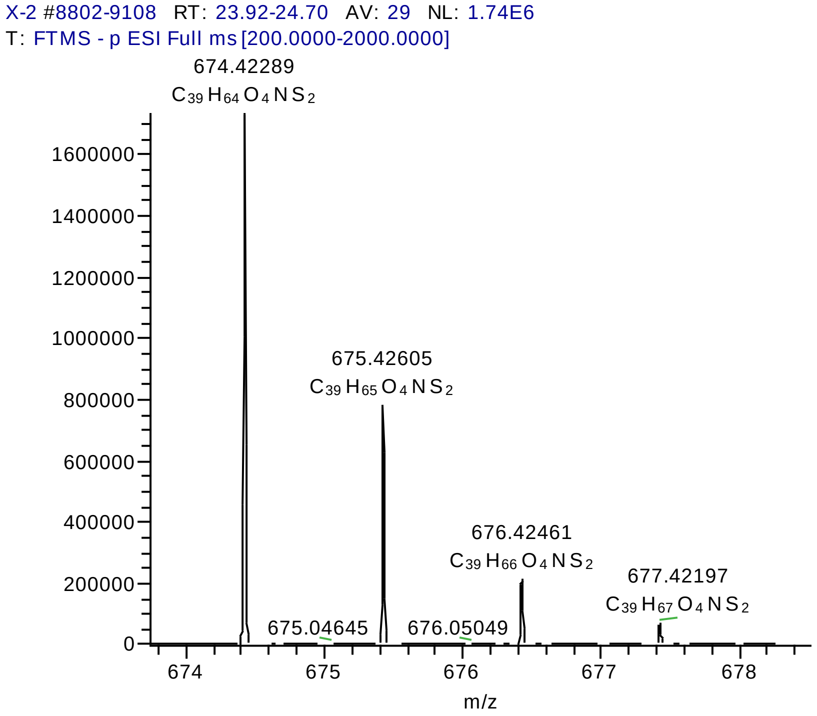

Figure S53. HRMS spectrum of 3j.

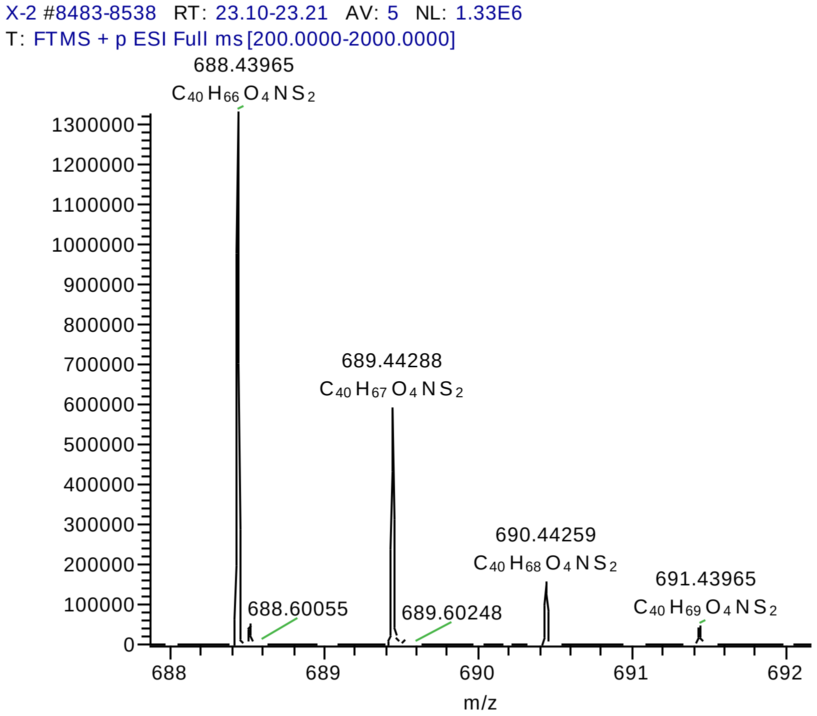

Figure S54. HRMS spectrum of 3k.

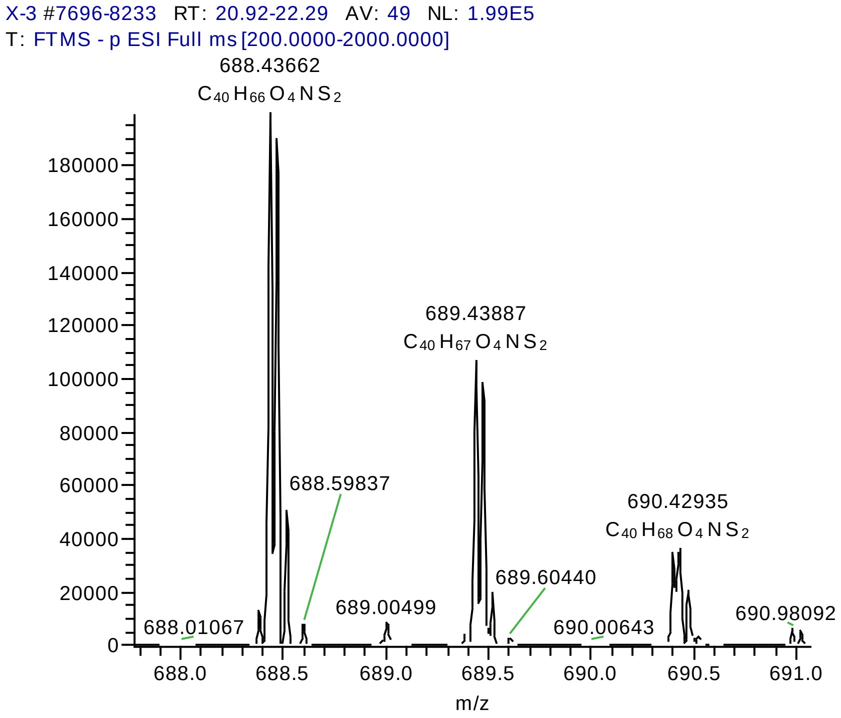

Figure S55. HRMS spectrum of 3l.

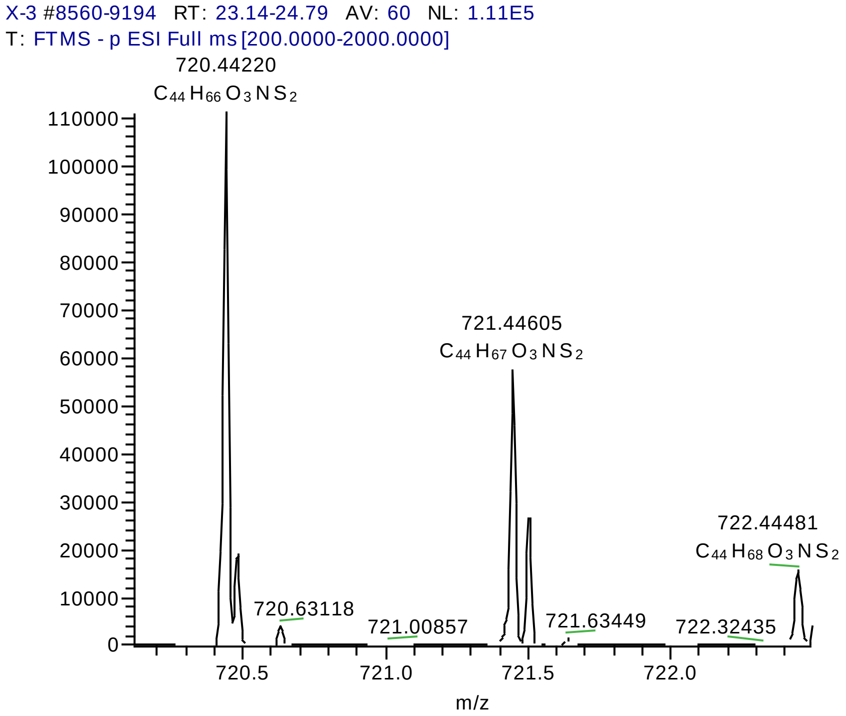

Figure S56. HRMS spectrum of 3m.

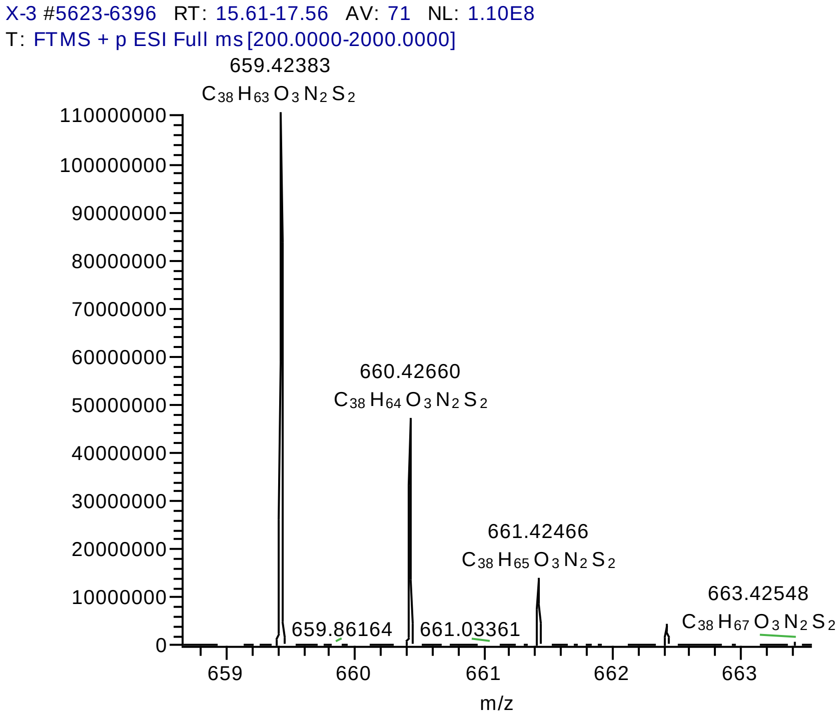

Figure S57. HRMS spectrum of 3n.

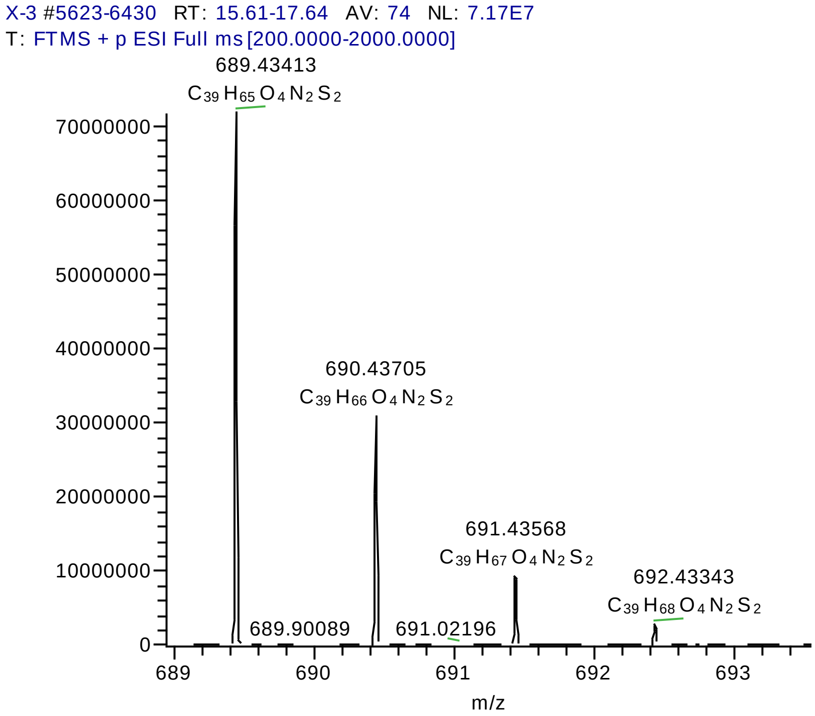

Figure S58. HRMS spectrum of 3o.

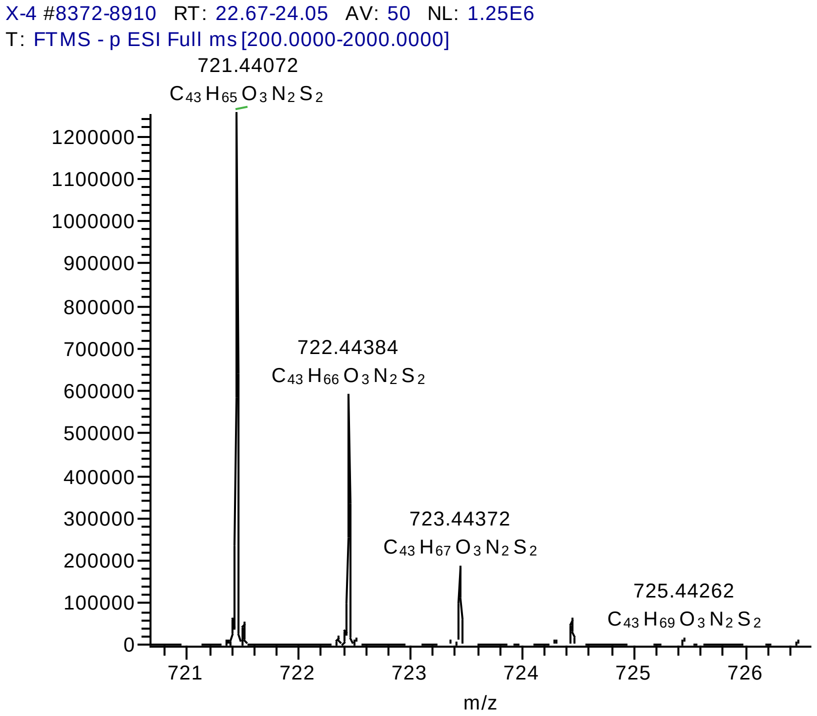

Figure S59. HRMS spectrum of 3p.

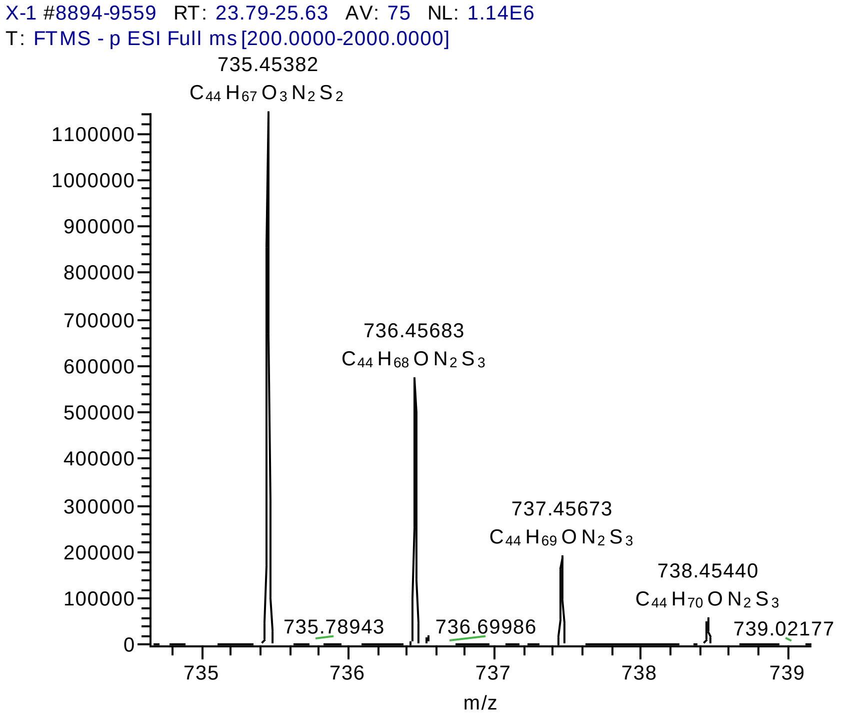

Figure S60. HRMS spectrum of 3q.

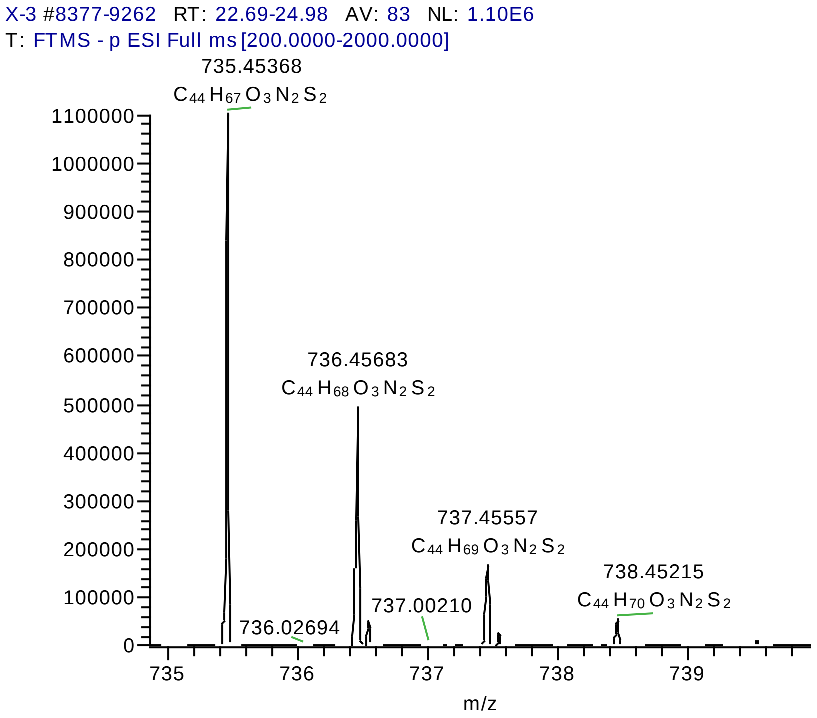

Figure S61. HRMS spectrum of 3r.

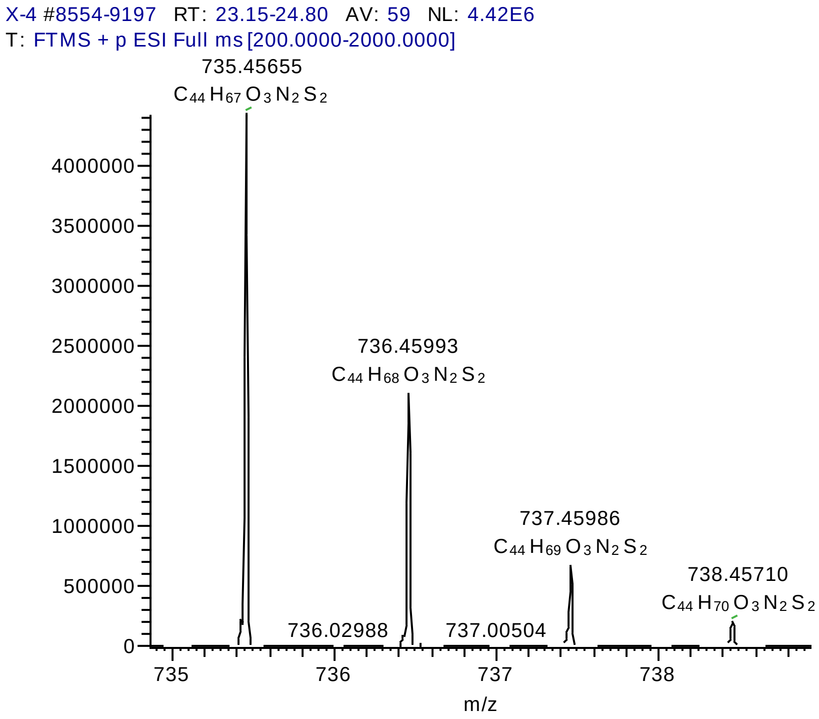

Figure S62. HRMS spectrum of 3s.

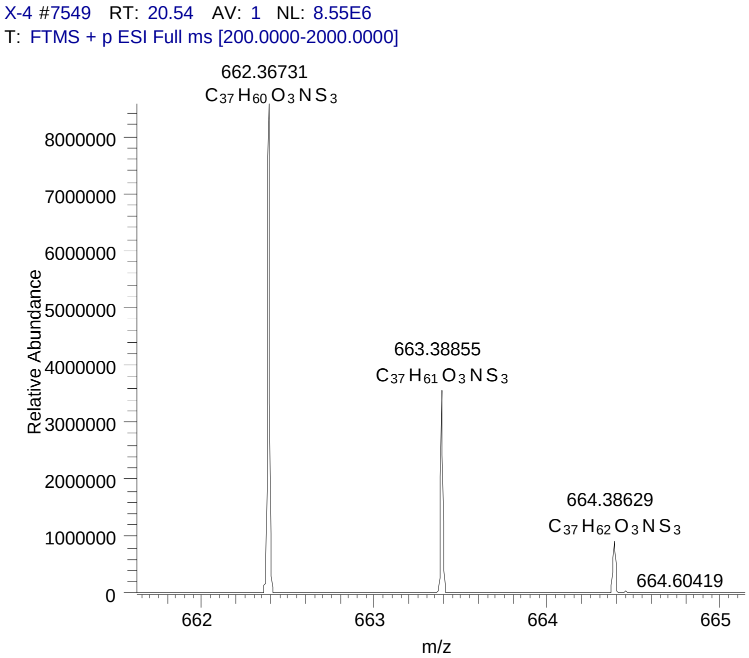

Figure S63. HRMS spectrum of 3t.
